# Nano-PhotoCrystals (Photosensitive Nanocrystals) of Aloe Emodin Combined with Dissolving Microneedles for Dual Smart Targeting of Acute Bacterial Skin and Soft Tissue Infections

**DOI:** 10.1021/acsami.5c18641

**Published:** 2025-11-20

**Authors:** Abraham M. Abraham, Qonita Kurnia Anjani, Eduard Preis, Yara A. Naser, Lena Bender, Franziska Bär, Udo Bakowsky, Elise Catlin, Alejandro J. Paredes, Lalitkumar K. Vora, Eneko Larrañeta, Ryan F. Donnelly

**Affiliations:** † School of Pharmacy, 1596 Queen’s University Belfast, Medical Biology Centre, 97 Lisburn Road, Belfast BT9 7BL, U.K.; ‡ Institute of Pharmaceutics and Biopharmaceutics, Department of Pharmacy, 9377 Philipps-Universität Marburg, Robert-Koch-Str. 4, 35037 Marburg, Germany

**Keywords:** acute bacterial skin and soft tissue infections, aloe emodin, *Aloe vera*, microneedles, PhotoCrystals, nanocrystals, transdermal drug delivery, photodynamic antibacterial therapy

## Abstract

Acute bacterial skin and soft tissue infections (ABSSTIs) are often treated with antibiotics, facing challenges with off-target toxicity and limited tissue penetration, with inadequate treatment escalating into serious conditions requiring hospitalization. Photodynamic antibacterial therapy (PACT), employing natural photosensitizers (PS), presents a promising alternative but is limited by poor water solubility, with modification strategies restricted by limited loading efficiency and drug release due to quenching effects (e.g., liposomes). To this end, this study explores the utilization of a matrix-free system, introducing the novel preparation of photosensitive nanocrystals (i.e., nanosized photosensitizers) referred to as nano-PhotoCrystals, derived from aloe emodin (AE), an anthraquinone naturally found in *Aloe vera*. Both nanosized and micronized AE PhotoCrystals were produced, with AE nanosized PhotoCrystals (N-PCs) prepared via bead-milling, and micronized AE PhotoCrystals (M-PCs) prepared by simple dispersion in surfactant solution were integrated with dissolving microneedles (MNs), to permit improved aqueous solubility and targeted dermal delivery. All formulations prepared were characterized for physicochemical properties, stability, and transdermal permeation and dermal deposition. It was found that N-PCs, with a mean particle size of approximately 200 nm, increased drug deposition in skin layers, achieving about 50 μg/cm^3^ in the dermis, while M-PCs, ∼40 μm in size, achieved less than 12 μg/cm^3^. Additionally, the incorporation of AE N-PCs within MNs further improved dermal drug delivery, demonstrating a well-improved deposition profile (>100 μg/cm^3^). Evaluation through a hen’s egg test-chorioallantoic membrane assay confirmed that AE N-PCs were biocompatible, with no irritation induced, promoting safe skin application. Significant PACT potential against *Staphylococcus saprophyticus* was observed, achieving over 3.8 log reduction in bacterial viability under irradiation with higher effectiveness compared to M-PCs. Thus, the developed PCs incorporated within MNs present a novel, effective strategy for MN-PACT dual targeting of ABSSTIs, addressing challenges in solubility and delivery for hydrophobic PSs while mitigating antimicrobial resistance concerns.

## Graphical abstract

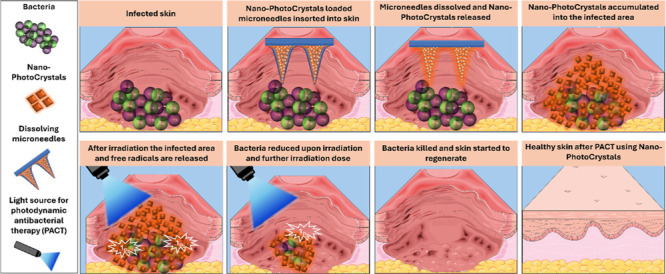

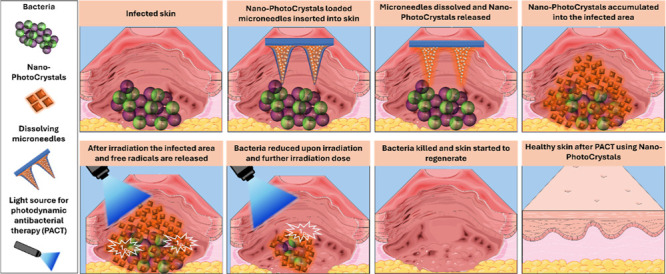

## 1 Introduction

Acute bacterial skin and soft tissue infections (ABSSTIs) are one of the most common infectious diseases, with approximately 16 million cases in the USA alone.^1,2^ ABSSTIs are caused by bacterial invasion of the varying skin layers and the deeper soft tissues, which can develop locally, forming tissue damage due to persistent recurring infections, and can progress to the bloodstream, resulting in life-threatening, difficult-to-treat conditions that require hospitalization with fatal complications leading to death. When the infectious microorganisms responsible for ABSSTIs reach the soft tissues of the skin, for example, in the case of skin cuts and wounds, the risk of such infections is higher and more difficult to treat. Furthermore, it has been reported that more than 40% of ABSSTIs cases admitted to the hospital required invasive intravenous antibiotic treatment or even long-term use of oral antibiotics, which are not without drawbacks.^2,3^ Such limitations include systemic off-target toxicity, detrimental disruption of the microbiome, reduction of microbial diversity, prolonged and potentially incomplete use of antibiotics threatening the emergence of resistance, as well as limited distribution locally to the skin and soft tissues.^4–6^ Hence, the poor blood perfusion within the infected area may limit the delivery of intravenously and orally administered antibiotics to the targeted tissue.^7^ To this end, designing an effective topical antibacterial delivery system capable of efficiently reaching deeper infected skin layers could be essential to tackling such infections, reducing the combined adverse effects and lowering the costs associated with intensive healthcare, thereby improving patient compliance and treatment outcomes. Precise targeting of the infected area is crucial to avoid disruption of the corresponding commensal microorganisms (i.e., the natural flora), prevent excessive antibiotic exposure, and avoid the unwanted development of antimicrobial resistance (AMR), which is considered one of the most challenging global health issues, particularly affecting low- and middle-income countries, according to the World Health Organization (WHO).^8^ AMR was responsible for more than a million deaths worldwide in 2019 alone and is projected to cause more than 10 million deaths annually by 2050.^9^ Therefore, there is an urgent need to develop localized antibacterial delivery strategies that can reduce the risk of antibiotic resistance while simultaneously addressing the issue of poor tissue perfusion in infected areas. One such strategy is photodynamic antibacterial therapy (PACT), which offers a promising alternative to conventional antibiotics by avoiding systemic side effects. PACT remains inactive in the dark and becomes therapeutically active only upon irradiation with light at an appropriate wavelength.^10^ This, in turn, permits precise treatment of the infected area and reduces possible side effects of the active pharmaceutical ingredient (API), thus mitigating AMR. The use of nontoxic and solubilized photosensitizers (PSs), a source of light, and molecular oxygen are prerequisites to effectively target microorganisms *via* PACT. After irradiation, the chosen PS transitions to a higher energy level (i.e., the excited state), leading to the generation of reactive oxygen species (ROS) that induce microbial cell death.^11,12^ Different PSs have been used in PACT, but recently, there has been great interest in using natural PSs instead of synthetic ones, providing a sustainable and eco-friendly source that generally exhibits fewer adverse effects.^13^ However, the potential of natural PSs is hindered by their intrinsic hydrophobicity, which limits their use in photodynamic therapy (PDT).^14,15^ A multitude of strategies have been employed to improve the poor water solubility of natural PSs, such as various cyclodextrin complexation techniques and nanocarriers (e.g., liposomes, lipid, and polymeric nanoparticles).^16–18^ However, these formulations are not without drawbacks, as they possess limited encapsulation efficiency, and the use of a matrix comprised of nonactive ingredients can affect drug release, leading to quenching and, thus, ineffective treatment.^5,19^ Therefore, a novel nanotechnological approach to improve the solubility of natural PSs without the need for matrix-based nanocarriers would broaden their applicability as promising tools in PDT.

Nanocrystals (NCs) can be utilized as a novel platform in the field of PDT, providing an improved dissolution profile and bioavailability for insoluble APIs.^20^ Furthermore, NCs are composed of 100% API without any matrix-based system, stabilized by a thin polymeric or surfactant coating, offering an increased surface area that makes them ideal for intradermal applications with improved skin permeation and deposition properties. This presents an encouraging approach to reducing the amount of API needed to reach an effective therapeutic level. The production of NCs using a top-down media milling approach is simple and scalable and permits NC production without expensive manufacturing techniques.^21^ To this end, reducing PSs to the nano-dimension, using a lab-scale benchtop method, to yield NCs for application in PDT, represents a novel approach to produce what has been termed here as photosensitive nanocrystals (i.e., nano-PhotoCrystals, N-PCs). This novel formulation principle improves the aqueous solubility of the PS and addresses the issues related to nanocarriers used in PDT. Due to their small size, the resulting N-PCs are expected to enhance the delivery of insoluble PS within the skin layers compared to larger particles, thus promoting greater PS availability for irradiation.

In this work, the objective was to present N-PCs as a novel, smart, and effective strategy in the PACT to treat ABSSTIs. As deposition of PS within the skin layers is essential for effective treatment, the current study aimed to combine the obtained N-PCs with noninvasive dissolving microneedles (MNs) to further improve local accumulation and transdermal delivery of the N-PCs into deep skin layers, thereby reaching the targeted underlying soft tissues. Dissolving MNs represent an innovative approach to enhance drug permeation through the skin and facilitate deposition within the skin layers.^22,23^ They feature an array of tiny needles, typically in the micrometer range, extending perpendicularly from a flat baseplate.^24,25^ When applied, these needles painlessly penetrate the *stratum corneum* (SC), creating temporary pathways for drug delivery without causing bleeding or contact with nociceptors, and in a minimally invasive manner.^23^ Dissolving MNs are made from a soluble and biodegradable matrix that dissolves upon skin application, releasing the drug components directly into the dermal layer.^26^ Hence, the drug is intradermally deposited or transdermally permeated.

In this work, aloe emodin (AE), a natural anthraquinone extracted from the root of *Aloe vera*, was chosen as a model natural PS.^27^ AE is known in Chinese medicine for its various pharmacological properties, including antibacterial activity as well as in different skincare products, such as treatments for dry skin.^28^ As such, exploration of AE’s phototoxicity in cosmeceutical products broadened its therapeutic scope with prospective use in PDT. To this end, the encapsulation of AE in different nanocarriers has been recently reported, presenting AE as a very promising PS candidate.^29,30^ To this purpose, AE nano-PCs produced using a bead-milling technique with different surfactants were compared with unmilled micro-PCs and further combined with dissolving MNs. All the formulations obtained were fully characterized regarding their physicochemical properties, stability, transdermal permeation, and dermal deposition with the PACT efficacy of the nanomilled AE (i.e., N-PCs) compared with that of unmilled micro-AE PhotoCrystals (M-PCs). Ultimately, to assess the biocompatibility and potential irritation of the obtained N-PCs and their MNs, a hen’s egg test-chorioallantoic membrane assay was utilized.

## 2 Materials and Methods

### 2.1 Materials

Aloe emodin (AE) (purity of 97%) was purchased from abcr GmbH (Karlsruhe, Germany). Tween 80 was purchased from Tokyo Chemical Industry (Oxford, UK) or Sigma-Aldrich (Dorset, UK). Poloxamer 188 (P188) was obtained from the BASF Chemical Company (Ludwigshafen, Germany). Zirconia beads partially stabilized with Yttria (type YTZP) with a diameter of 0.1–0.2 mm (small beads) and Yttrium stabilized zirconium oxide beads with a diameter of 1–1.2 mm (bigbeads) were obtained from Chemco (Guangfu, China). Poly­(vinylpyrrolidone) K29–32 (PVP) with a molecular weight of 58 kDa was provided by Ashland (Kidderminster, UK). Poly­(vinyl alcohol) (PVA) (*M*_W_) 9–10 kDa, PVA (*M*_W_ 31–50 kDa, 80% hydrolyzed), and methanol 99% (v/v) were purchased from Sigma-Aldrich (Dorset, UK). Ultrapure water was obtained from a water purification system [high-performance liquid chromatography (HPLC) grade, Elga PURELAB DV 25, Veolia Water Systems, Dublin, Ireland] or from PURELAB flex 4 (ELGA LabWater, High Wycombe, UK). Liquid silicone rubber (DDR-4320) was obtained from Nusil Technology (Buckinghamshire, UK). *Staphylococcus saprophyticus*subsp.*bovis* (*S. saprophyticus*, DSM no. 18669) and *Escherichia coli* DH5 alpha (*E. coli*, DSM no. 6897) were obtained from DSMZ (Braunschweig, Germany). Fertilized eggs of *Gallus domesticus* were obtained from a hatchery Brormann GmbH & Co. KG (Rheda-Wiedenbrück, Germany). Isopropanol (99.9%) was obtained from Sigma-Aldrich (Taufkirchen, Germany). Sterile scalpels were purchased from B.Braun (Melsungen, Germany), and Dumostar Style 7 curved tweezers were obtained from Dumont (Montignez, Switzerland). Irradiation was performed with the Weberneedle Endolaser in combination with a Weberneedle Lasercath (Weber Medical, Lauenförde, Germany).

### 2.2 Methods

#### 2.2.1 Production of AE M-PCs and N-PCs

AE N-PCs were prepared from unmilled AE M-PCs. AE M-PCs were produced by admixing 10% (w/w) unprocessed AE powder and surfactant (Tween 80, Poloxamer 188 or a 1:1 mixture of PVA 9–10 kDa and PVP K29–32). Subsequently, the N-PCs were prepared using small-scale bead milling (with a bead/suspension ratio of 40:60 (v/v)).^31^ The milling chamber was a 25 mL Erlenmeyer flask containing Yttrium-stabilized zirconium oxide beads as milling medium and a triangle magnetic stirring rod (ASTEROID 25, 2mag AG, München, Germany) as a milling shaft. The milling media and pearls were then rotated by using a magnetic stirrer (IKA-Combimag RCT, IKA, Staufen, Germany) at 1500 rpm for 24 h. Surfactant screening was performed for the three surfactants based on the milling time and the concentration of the surfactant used while keeping all other parameters the same. To this end, milling times of 1, 3, 6, 12, and 24 h were tested, and three different concentrations of 0.5, 1, and 2% w/w of each of the formerly mentioned surfactants were used. Throughout the milling process, the samples were protected from light and kept on an ice bath.

To remove water from the formulated AE PCs, thereby producing concentrated AE nano- and micro-PhotoCrystal powder capable of redispersion, lyophilization was employed. Samples composed of AE N-PCs and AE M-PCs were lyophilized using the previously described method by McKenna et al.^26^ Briefly, samples were frozen at −80 °C for 10 h before lyophilization and then lyophilized in a Virtis Advantage Benchtop Freeze Drier (SP Scientific Ltd., Warminster, PA). The following freeze-drying parameters were used: primary drying at −40 °C for 90 min, followed by 90 min at −30 °C, then for 90 min at −20 °C, increasing to −10 °C for 530 min, and finally for 90 min at 0–10 °C. The secondary drying step was composed of 660 min at a room temperature of 25 °C. A vacuum pressure of 50 mTorr was maintained throughout the entire process.

#### 2.2.2 Characterization of AE M-PCs and N-PCs

##### 2.2.2.1 AE M-PCs and N-PCs Particle Size and Distribution

Dynamic light scattering (DLS) and static light scattering (SLS; also referred to as laser diffraction, LD) as two independent methods were used to determine the particle size and homogeneity of the N-PCs and the M-PCs.^32^ DLS (NanoBrook Omni Particle size and zeta potential analyzer, Brookhaven Instruments Corporation, Holtsville, USA) was used to determine the N-PCs size, with regard to its hydrodynamic diameter, and the polydispersity index (PDI) as a measure for the width of the size distribution. Measurements were performed in at least triplicate; the measuring conditions were adjusted to 20 °C. The data were analyzed with the general-purpose mode built into the software of the instrument. Furthermore, SLS (Mastersizer 3000, Malvern Panalytical Ltd., Malvern, UK) was used to measure any remaining large particles/agglomerations after the nanomilling and to characterize the M-PCs.^33^ SLS data analysis was determined by application of the Mie-theory, with optical parameters set to 1.50 for the real refractive index and 0.1/0.01 for the blue light (470 nm) and the red light (633 nm) imaginary refractive indices.^21^ SLS data were presented as median volume-based diameters (d­(*v*) 0.1 – d­(*v*) 0.90). The particle size and size distribution of AE N-PCs after freeze-drying were determined and compared to those of fresh AE N-PCs, to determine if the process of freeze-drying led to particle aggregation and therefore stability issues.

##### 2.2.2.2 Zeta Potential Analysis of AE N-PCs

The Zeta potential (ZP) is a crucial parameter for predicting the physical stability of nanoformulations, as it represents the electric potential at the slipping plane of the electrical double layer.^31^ The ZP was determined by measuring the electrophoretic mobility using a Doppler anemometry (LDA) (NanoBrook Omni Particle size and zeta potential analyzer) and the use of the Helmholtz–Smoluchowski equation. Measurements were attained in conductivity-adjusted purified water (50 μS/cm/20 °C) and in the original medium (i.e., surfactant solutions). The analysis was performed in at least triplicate and reported as an average ± standard deviation (SD).

##### 2.2.2.3 Visualization of AE M-PCs and N-PCs

Freshly prepared AE N-PCs were visualized by using transmission electron microscopy (TEM) (JEM-1400Plus; JEOL, Tokyo, Japan). The N-PCs were diluted with water (∼100-fold), followed by vortexing for 5 min to ensure homogeneous mixing. Subsequently, a drop of the diluted sample was precisely placed on a copper grid and left to dry at room temperature prior to capturing the images. Pure AE and the M-PCs were visualized using scanning electron microscopy (SEM) with a Hitachi TM3030 tabletop SEM microscope (Chiyoda-ku, Tokyo, Japan). This was performed under a low vacuum at an excitation voltage of 15 kV and a 1000× magnification.

##### 2.2.2.4 Determination of the Crystallinity State Using X-ray Diffraction (X-RD) of AE M-PCs and N-PCs

X-RD analysis of pure AE, AE M-PCs, and N-PCs produced was obtained using a MiniFlex II powder X-ray diffractometer with PDWL software (Rigaku Corporation, Tokyo, Japan). Patterns were collected in continuous mode in the angular range of 3–45° 2θ, with a step size of 0.01°, a scanning rate of 2°/min, a voltage of 30 kV, and a current of 15 mA.

##### 2.2.2.5 Differential Scanning Calorimetry of AE M-PCs and N-PCs

Differential scanning calorimetry (DSC) analysis was performed on samples of pure AE, AE M-PCs, and N-PCs prepared using an Advantage Model Q100 DSC (TA Instruments, New Castle, DE). Samples of 3–15 mg were weighed accurately, placed in aluminum pans that were then sealed by crimping, and subsequently heated at a rate of 10 °C per min from 30 to 250 °C under a nitrogen flow of 50 mL/min.

##### 2.2.2.6 Attenuated Total Reflection Fourier Transform Infrared of AE M-PCs and N-PCs

Attenuated total reflection Fourier transform infrared (FTIR) spectroscopy was used to analyze interactions present in pure AE, AE M-PCs, and N-PCs produced using an Accutrac FT/IR-4100 Series (Jasco, Essex, U.K.) equipped with MIRacle diamond ATR accessory (Pike Technologies Ltd., Madison, WI). The IR spectra were scanned and recorded in the region ranging from 4000 to 600 cm^–1^ at room temperature. Resolution was maintained at 4.0 cm^–1^ throughout the analysis, with the resultant spectra obtained from an average of 64 scans.

##### 2.2.2.7 In Vitro Dissolution Release Study of AE M-PCs and N-PCs

In vitro dissolution release of the AE resuspended freeze-dried N-PCs was performed using a dialysis bag method, previously described by Freag et al.^34^ Both pure AE (suspended in water) and AE resuspended freeze-dried M-PCs were used as controls, with each sample (equivalent to 2.5 mg AE) added into a sealed cellulosic dialysis membrane tubing (Spectra-Por4 dialysis tubing, cut off 12–14 kDa, Spectrum Laboratories Inc., New Brunswick, NJ, USA). The dialysis bags were placed in 200 mL of the release medium composed of phosphate buffer, pH 7.4, containing 1% w/w Tween 80 to maintain sink conditions and incubated at 37 °C ± 0.5 °C while being shaken at 40 rpm. 200 μL aliquots were withdrawn at predetermined time points of 1, 2, 3, 4, 5, 6, 7, 8, 24, and 48 h and replaced with the same volume of fresh release medium. The samples were then filtered using a 0.45 μm Millipore filter (Millipore; Thermo Fisher Scientific, Waltham, MA, USA) and quantified using a developed HPLC method (cf. Section 2.2.4.4).

#### 2.2.3 Production of AE PCs-Loaded Dissolving Microneedles

Dissolving microneedles (MNs) of AE M-PCs and N-PCs were produced using the micro molding method, via a two-layer casting approach, as previously described by Anjani et al.^35^ The composition of aqueous polymer blends utilized in each layer is shown in Table 1. With the formulation of the first layer composed of resuspended AE M-PCs and AE N-PCs using either 10% or 40% w/w of the polymers used and a second casting of a drug-free baseplate corresponding to the polymers shown in Table 1 to cast the second layer. Two different molecular weights (*M*_W_) of PVA were selected for the needle and baseplate regions, as this parameter influences both the mechanical strength and dissolution rate. To achieve optimal performance, a bilayer MN design was employed: low-*M*_W_ PVA (9–10 kDa) in the needle region enabled efficient mold filling, rapid dissolution, and effective drug release, while high-*M*_W_ PVA (31–50 kDa) in the baseplate provided mechanical integrity and ease of handling.^36^ A previously developed silicone mold housing a 16 × 16 array of preformed microneedle cavities (each microneedle tip exhibited a pyramidal geometry with a height of 600 μm, anchored on a cuboidal base measuring 250 μm in width, with a basal width of 300 μm and an interspacing of 150 μm) and a total needle height of 850 μm was used.

**Table 1:** Formulation Used to Obtain the Dissolving MNs Cast in a Two-Step Approach (First and Second Layer) Showing the Polymers Used and Their Corresponding Molecular Weight Expressed by kiloDalton (kDa)

MNs layer	polymer	molecular weight (kDa)	ratio of the used polymers
first layer (needles)	PVP: poly(vinylpyrrolidone) K29–32	58	1:1
	PVA: poly(vinyl alcohol)	9–10	
second layer (baseplate)	PVP: poly(vinylpyrrolidone) K29–32	58	1:1
	PVA: poly(vinyl alcohol)	31–50	

To study the effects of loading the M-PCs and N-PCs in the MNs, the size and ZP of the PCs were measured (cf. Sections 2.2.2.1 and 2.2.2.2) by dissolving the MNs in an appropriate volume (5 mL) of ultrapure water, followed by five centrifugation-washing cycles to remove the polymers. The remaining sample was dried at room temperature, with the obtained powder resuspended in water prior to analysis.

#### 2.2.4 Characterization of AE PCs-Loaded Dissolving MNs

##### 2.2.4.1 Mechanical Properties of PCs-Loaded Dissolving MNs

In order to evaluate the compression resistance of the AE PhotoCrystal-loaded dissolving MNs, a TA.XT.Plus Texture Analyzer (Stable Micro Systems, Surrey, UK) was utilized in compression mode, as described in previous work.^24^ The baseplate of the dissolving MNs was positioned on the aluminum probe with the needles facing the aluminum block. The Texture Analyzer settings were adjusted to a pre-test speed, test speed, and post-test speed of 10, 0.5, and 10 mm/s, respectively. The height of the microneedle arrays was measured before and after testing using a digital light microscope (Leica Microsystems, Milton Keynes, Buckinghamshire, UK), and the percentage MN height reduction was calculated according to eq 1

1 $$needle\;height\;reduction\;\left(\%\right)=\left(\frac{H_{b}-H_{a}}{H_{b}}\right)\times 100$$

where *H*_b_ represents the initial height prior to compression and *H*_a_ represents the final height postcompression.

##### 2.2.4.2 Insertion Profile of AE PCs-Loaded Dissolving MNs

The insertion efficiency of the M-PCs and N-PCs MNs was assessed, using a Texture Analyzer and an artificial skin model consisting of eight layers of Parafilm M, following a protocol detailed by Larrañeta et al.^24^ Insertion efficiency was determined using eq 2

2 $$insertion\;efficiency\;in\;the\;{Parafilm}^{®}\;M\;\left(\%\right)=\left(\frac{number\;of\;holes\;counted}{number\;of\;micro\;needles\;in\;an\;array}\right)\times 100$$

Furthermore, the insertion properties of the MNs into the Parafilm M skin model were analyzed using an EX-101 optical coherence tomography (OCT) microscope (Michelson Diagnostics Ltd., Kent, UK), whereby images were captured to visualize the depth of needle insertion and ImageJ software (National Institutes of Health, Bethesda, MD) was used to calculate the exact depth of insertion.

To further characterize the insertion properties of the MNs, the MNs were inserted into full-thickness neonatal porcine skin, following the method previously described, with subsequent visualization using an OCT microscope and ImageJ software to determine the height of needle insertion.^35^

##### 2.2.4.3 Visualization of AE PCs-Loaded Dissolving MNs

Images of M-PCs and N-PCs MNs were obtained using a light microscope with an affixed color camera (Leica Microsystems, Milton Keynes, Buckinghamshire, UK) to confirm complete needle formation with sharp and intact needles and the absence of drug distribution in the baseplate after casting the second layer (i.e., drug-free baseplate). Furthermore, to provide a more detailed view of the needle’s structure, SEM images were captured under low vacuum conditions with an excitation voltage of 15 kV and a 1000× magnification, using a Hitachi TM3030 tabletop SEM microscope (Chiyoda-ku, Tokyo, Japan).

##### 2.2.4.4 Chemical Analysis and Drug Content of AE PCs-Loaded Dissolving MNs

AE content was determined using a previously reported HPLC method.^37^ Briefly, an Agilent Technologies 1220 infinity compacted LC series, including degasser, binary pump, auto injector, and UV detector at 256 nm (Agilent Technologies UK Ltd., Stockport, UK), was used. A HPLC column (Phenomenex Luna C18 (ODS1), 150 × 4.6 mm internal diameter, 5 μm packing) was obtained from Phenomenex (Cheshire, UK) and used to separate AE from a mixture of compounds. The analysis was performed at room temperature by injecting 25 μL of the sample at a flow rate of 0.5 mL/min and a run time of 13 min. The mobile phase consisted of 0.1% (v/v) formic acid, water, and methanol (30:70 v/v). The chromatograms were analyzed using the Agilent ChemStation Software B.02.01. The International Council of Harmonization 2005 guidelines were followed as a reference to validate all the analytical methods.

For drug content quantification, MN array patches were dissolved in 5 mL of ultrapure water and sonicated for 30 min to dissolve the hydrophilic polymer. The mixture was added to 5 mL of methanol and sonicated for 30 min. The obtained sample was centrifuged at 14,500 rpm for 15 min, with the supernatant removed, and subjected to HPLC analysis.

##### 2.2.4.5 In Situ Dissolution Study of AE PCs-Loaded Dissolving MNs

Prior to the dissolution study, excised full-thickness neonatal porcine skin was shaved and soaked in a phosphate buffer saline (PBS) solution with a pH of 7.4 for 30 min at room temperature. The selected MN arrays were inserted into the skin using manual thumb pressure for 30 s, with 5.0 g cylindrical stainless-steel weights placed on top of each of the MNs to prevent the MNs array patch from being pushed out during the study. The samples were kept at 37 °C for 30 and 60 min, and the MNs were subsequently removed from the skin, with the morphology of the needles and the punctured porcine skin examined under a digital light microscope.

##### 2.2.4.6 AE PCs Characterization after Incorporating in Dissolving MNs

M-PCs and N-PCs incorporated with MNs were analyzed to confirm the properties of AE after the energy-intensive milling process and the suitability of incorporating within the chosen polymers required to fabricate the MNs. For this, the MNs were dissolved using 5 mL of ultrapure water and then centrifuged at 5000 rpm for 15 min. The supernatant was removed, and the obtained pellet containing AE was resuspended, washed, and centrifuged at 5000 rpm for 15 min with ultrapure water five times. The obtained pellet was dried at room temperature and protected from the light. Following this, the particle size was measured using DLS and SLS, as described in Section 2.2.2.1. Furthermore, the ZP was determined to identify any potential surface modifications that may interact with the polymers used to fabricate the MNs (cf. Section 2.2.2.2). X-RD, DSC, and FTIR (cf. 2.2.2.4, 2.2.2.5 and 2.2.2.6) were also performed and compared with the physical mixture of AE-MNs (prepared by dry mixing pure AE with the polymers used for MN fabrication) to further confirm if the chemical properties were maintained after MN production and to ensure the integrity of AE, to enable effective release upon the solvation of the dissolving MNs.

MNs were used for all of the aforementioned tests except X-RD. For this, an equivalent film of 5 × 5 cm^2^ with the same composition and concentration as the MNs was produced and exposed to the same manufacturing conditions applied to the MNs. This was due to the larger sample size required to obtain reliable data using X-RD.

##### 2.2.4.7 Stability Study of AE PCs-Loaded Dissolving MNs

M-PCs and N-PCs MNs were subjected to a stability study under standard and accelerated conditions. Thereby, the MNs stored under accelerated conditions were kept at 40 °C at 75% relative humidity for 1 month. Meanwhile, a 3-month stability study was performed under standard conditions, where the MNs were stored at 25 °C at 65% relative humidity. All the samples used for the stability study were analyzed regarding their mechanical properties (cf. 2.2.4.1) and AE content (cf. 2.2.4.4).

#### 2.2.5 In Vitro Skin Permeation and Penetration Study of AE Formulations

A transdermal permeation profile of AE M-PCs and N-PCs incorporated into the MNs was achieved using the Franz cell apparatus (PermeGear, PA, USA) and compared to the penetration profile obtained of AE from the M-PCs and N-PCs (i.e., in the absence of the MNs), respectively. An equivalent amount of freeze-dried AE M-PCs and N-PCs to that loaded within the MNs was resuspended in ultrapure water, applied to the porcine skin using a pipet, and spread carefully using a saturated glove with the corresponding formulation. An alcoholic solution containing the same quantity of AE loaded within the MNs (based on the drug content analysis) was used as a control. Full-thickness neonatal porcine skin was shaved and affixed on the top of the donor compartment of Franz cells and glued with cyanoacrylate glue (Stick it super glue, PLDZ Pattison House, Dublin, Ireland) with the subcutaneous side facing the receiver compartment. The MNs were manually inserted into the skin, using a 5 mL syringe plunger, with firm pressure applied for ∼30 s. A cylindrical stainless-steel weight (12 g) was placed on top of each MN to retain its position within the skin. The donor compartments were placed onto the receiver compartments, each containing 12 mL of PBS (pH 7.4) with 2% w/v Tween 80 acting as a release medium in order to achieve the sink conditions required.^34^ Both the stirring and temperature remained constant throughout the experiment at 600 rpm and 37 ± 1 °C, respectively. Each individual Franz diffusion cell was sealed with Parafilm M to prevent evaporation and covered with aluminum foil to protect the cells from light and thus prevent the degradation of AE. At specific time points (0, 1, 2, 3, 4, 5, 6, 8, 10, 12, and 24 h), samples were taken and replaced with the same amount of the release medium.

#### 2.2.6 In Vitro Skin Deposition Study of AE Formulations

In order to quantify the AE deposited within the skin layers during the in vitro transdermal studies, it was essential to develop a method to ensure complete extraction of AE from the skin.^24^ As such, the epidermis and dermis of the application areas (∼1 cm^2^) were separated using the previously described protocol.^38^ Initially, the skin was cleaned with a PBS-wetted tissue and heated in a weighing boat at 80 °C, with removal of the epidermal layer using a spatula and subsequent collection in a 2 mL Eppendorf tube. 0.5 mL of ultrapure water was added to remove any remaining polymers of the MNs and to ensure identical conditions; this step was adhered to all formulations. The samples were shaken at 1500 rpm for 1 h at room temperature using an Eppendorf ThermoMixer C. After this, 1.5 mL of methanol was added and shaken at 1500 rpm for 3 h at room temperature using an Eppendorf ThermoMixer C.

The remaining part of the skin, comprised of the dermis, was sectioned into small pieces using scissors and then placed into 2 mL Eppendorf tubes with two metal beads. Samples were subjected to a homogenization cycle in 1000 μL of methanol using Tissue Lyser LT (Qiagen Ltd., Manchester, UK) with two 5 mm stainless steel beads (Qiagen, Hilden, Germany) at 50 Hz for 15 min. 500 μL of methanol was added, and a homogenization cycle utilizing the same parameters was repeated. Samples were transferred to a 15 mL Falcon tube, with 8.5 mL of methanol added, and sonicated overnight in an environment protected from light.

Ultimately, all the samples were centrifuged at 15,000 rpm for 15 min, and the supernatant was collected to determine the amount of AE within the skin layers (i.e., epidermis and dermis) using the HPLC method explained previously (cf. 2.2.4.4).

#### 2.2.7 Photodynamic Antibacterial Therapy of AE M-PCs, N-PCs, and Their MNs

##### 2.2.7.1 Bacterial Strains and Media

*S. saprophyticus* subsp.*bovis* (DSM no. 18669) and *E. coli* DH5 alpha (DSM no. 6897) were sourced from DSMZ (Braunschweig, Germany) and stored as glycerol stock cultures at −80 °C. Prior to the bacterial viability assay, the stock culture was thawed and grown overnight in Mueller Hinton broth (MHB, Sigma-Aldrich Chemie GmbH) using an orbital shaker (Compact Shaker KS 15 A, equipped with Incubator Hood (TH 15, Edmund Bühler GmbH, Bodelshausen, Germany) set at 200 rpm and 37 °C.

##### 2.2.7.2 Light Source

The irradiation experiments were conducted using a specially designed light-emitting diode (LED) device (Lumundus GmbH, Eisenach, Germany), as described in a previous study by Preis et al.^39^ The device featured multiple arrays of LEDs arranged in parallel within a metal casing covered by transparent glass, ideal for irradiating various multiwell plates. Settings for irradiation time, current, and wavelength could be easily adjusted depending on the user preference.

##### 2.2.7.3 Photodynamic Antibacterial Therapy

Both *S. saprophyticus*subsp.*bovis* and *E. coli* DH5 alpha were treated with equal bacterial densities. The overnight culture of each organism was measured for optical density (OD_600_) using a spectrophotometer (Shimadzu UV mini-1240, Kyoto, Japan) and diluted appropriately to an OD_600_ of 0.025.^39^ The diluted suspension was incubated in an orbital shaker (Compact Shaker KS 15 A, equipped with Incubator Hood TH 15, Edmund Bühler GmbH) at 300 rpm and 37 °C until an OD_600_ of 0.400. To prevent further growth, the bacterial suspension was kept on ice; 250 μL of the bacterial suspension and 250 μL of AE M-PCs or N-PCs were mixed, and three different concentrations were evaluated (1, 4, and 16 μg/mL AE). The corresponding MNs were placed in a 12-well plate, PBS (pH 7.4) was added, and the samples were incubated in an orbital shaker at 150 rpm and 37 °C. 8 μL of the dissolved samples were mixed with 492 μL of bacterial suspension, resulting in a total AE concentration of 4 μg/mL. The mixtures were transferred to another 12-well plate and incubated at room temperature for 30 min, shielded from light. The samples were irradiated using the described LED device for 10 min at λ = 457 nm in 12-well cell culture plates (TC plate, Standard, F, Nümbrecht, Germany), with a radiant exposure of 13.2 J/cm^2^.^39^ After irradiation, the samples were serially diluted and plated onto Mueller Hinton II Agar plates (BD, Heidelberg, Germany). The agar plates were incubated for approximately 16 h at 37 °C under a relative humidity of 90% (In-VitroCell ES NU-5841E, NuAire, Inc., Plymouth, MN, USA), with the colonies counted to calculate the colony-forming units per milliliter (CFU/mL). Surfactant solution and blank MNs were used as the vehicle controls, while PBS (pH 7.4) served as a negative control, with dark controls performed for all samples.

#### 2.2.8 Hen’s Egg Test-Chorioallantoic Membrane Assay

The chorioallantoic membrane (CAM) forms from the fusion of the chorion and the vascularized allantoic membrane in a fertilized hen’s egg. *Gallus domesticus* eggs from the Brormann hatchery in Germany were utilized for the study. Prior to cultivation, the eggs were cleaned and disinfected with 70% isopropanol and placed in a hatching incubator set at 37 °C under 65% relative humidity. After embryonic development day (EDD) 3, the eggs were rotated 12 times per day while being positioned horizontally. On EDD 3, the eggs were opened using a pneumatic egg puncher with a diameter of 26 mm (Schuett-Biotec GmbH, Göttingen, Germany), sterile scalpels (B.Braun, Melsungen, Germany), and Dumostar Style 7 curved tweezers (Dumont, Montignez, Switzerland) and covered with Petri dishes before being returned to the incubator vertically for further experiments.

The hen's egg test-chorioallantoic membrane (HET-CAM) assay was used to evaluate the irritant potential and biocompatibility of the AE formulations. A volume of 0.3 mL of each formulation and control samples was applied to the CAM and observed for 5 min using a stereomicroscope. A positive control of sodium hydroxide and a negative control of normal saline were also used. Micrographs of reactions such as hemorrhage, lysis, and coagulation were captured with a digital camera Nikon Z6 (Nikon Europe BV, Amstelveen, Netherlands) connected to the microscope. Images of the same egg were captured at 0 min and after 5 min using an identical camera setup and, as closely as possible, the same positioning.

The irritation score (IS) was calculated following the Budai et al. method,^40^ as to assess the irritation potential and biocompatibility based on observed time points. Equation 3 was used to calculate the irritation score (IS)

3 $$IS=\frac{301-H_{\left[s\right]}}{300}\times 5+\frac{301-L_{\left[s\right]}}{300}\times 7+\frac{301-C_{\left[s\right]}}{300}\times 9$$

where *H* is hemorrhage, *L* is lysis, and *C* is coagulation expressed over time in seconds (s) when the phenomenon started.

#### 2.2.9 Statistical Analysis

At a minimum, all experiments were performed in triplicate. GraphPad Prism 10 (GraphPad Software, San Diego, CA) was used for statistical analysis. Where appropriate, an unpaired *t*-test was used for comparison of two groups. A one-way analysis of variance (ANOVA) and posthoc tests with Tukey correction and two-way ANOVA were used for the comparison of multiple groups. In all cases, data were reported as the mean ± standard deviation or the mean + standard deviation. *P*-values <0.05 were denoted as statistically significant.

## 3 Results and Discussion

### 3.1 Characterization of AE PCs

#### 3.1.1 Physicochemical Characterization of AE PCs

AE N-PCs were produced by using a small-scale wet bead milling method. For this, two types of beads were studied in terms of the milling efficiency. Samples were continuously drawn during the AE milling process for particle size analysis. The characteristics of the obtained samples were studied using DLS and LD, the surface charge using ZP, morphology using TEM, with the crystallinity and chemical interactions using DSC, X-RD, and FTIR. Optimal milling times and stabilizing effects on AE PCs were investigated for each formulation, with milling ceased after constant mean particle sizes and polydispersity indexes were obtained.

DLS and LD data were in agreement, highlighting that smaller particle sizes were achieved after utilization of the small beads during milling compared to the larger beads (Figure 1A,B). Although the difference was determined not to be significantly different, all milling processes performed thereafter used the smaller beads. Furthermore, there was no significant difference in the ZP values [in the ultrapure water and the original milling media (i.e., the surfactant solution)] of the PCs obtained by the small and big beads (Figure 1C). Additionally, the use of Tween 80 showed similar results to the data previously reported that the small beads are more efficient in producing smaller particles.^41^ However, more detailed surfactant screening using Tween 80, Poloxamer 188, and PVA/PVP was also performed. These three surfactants were screened with regard to the milling time (1–24 h) and concentration (0.5–2%).

**Figure 1:**
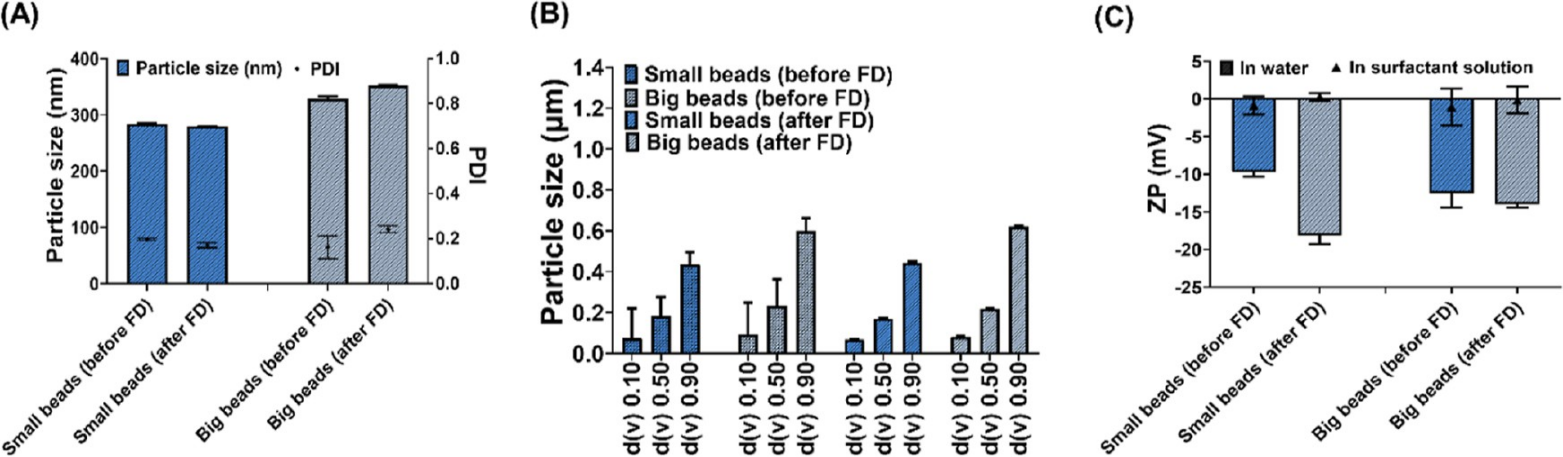
Physicochemical characterization of PCs nanomilled using small beads and big beads before and after FD (freeze-drying). (A) DLS data expressed as particle size in nm and PDI. (B) Laser diffraction data showing particle size expressed in μm. (C) ZP (zeta potential) values measured in water and surfactant solution. Data presented as means ± SD or means + SD, *n* = 4.

DLS data showed big particles after 1 h that are in the >2000 nm range for all the screened surfactants, highlighting further milling would be required to reach the average particle size of NCs typically employed within drug delivery systems. After 2 h, the DLS data show that the particle size reduced to approximately 300 nm in all the different concentrations. Interestingly, increasing the milling time for the three surfactants did not show significant changes in the particle size but resulted in more homogeneous particles, indicated by the relatively narrow size distribution, denoted by a PDI of <0.2 (Figure 2A,C,E). As evidenced within literature, PDI values below 0.2 are deemed suitable and indicate a uniform monodisperse suspension, thus confirming a narrow size distribution, agreeable for pharmaceutical applications, could be achieved in a relatively short period of time.^42^ N-PCs obtained with Tween 80 and Poloxamer 188 (highest concentration 2% w/w), milled for 24 h, produced the smallest particle size (approximately 200 nm) with desirable PDIs less than 0.2 (Figure 2C). Using PVA/PVP led to evidently larger particles than Tween 80, with DLS mean diameters of approximately 500 nm (Figure 2A). Additionally, the PDI was more than 0.2, revealing a more heterogeneous particle size distribution, promoting the potential of Ostwald ripening.^43,44^ However, a limitation of this technique is that it cannot detect particles larger than 10 μm.^45^ As a result, additional methods are necessary to identify any larger particles remaining after nanosizing. Thus, to overcome this, a low-angle SLS (i.e., LD) was used for detecting larger particles.

**Figure 2:**
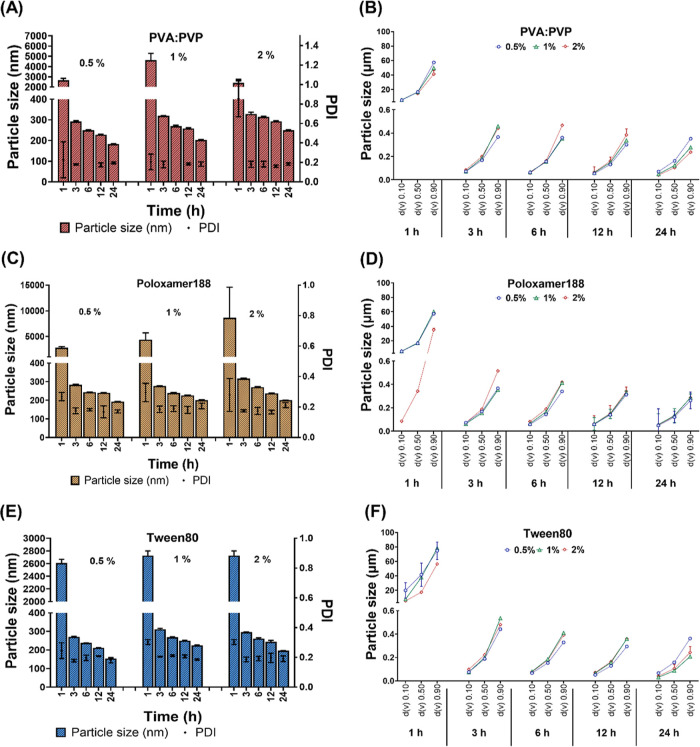
Physicochemical characterization of PCs nanomilled using PVA/PVP (poly­(vinyl alcohol)/poly­(vinylpyrrolidone) K29–32) as surfactant, showing (A) DLS data expressed as particle size in nm and PDI and (B) laser diffraction data showing particle size expressed in μm. Physicochemical characterization of PCs nanomilled using Poloxamer 188 as surfactant, showing (C) DLS data expressed as particle size in nm and PDI and (D) laser diffraction data showing particle size expressed in μm. Physicochemical characterization of PCs nanomilled using Tween 80 as surfactant, showing (E) DLS data expressed as particle size in nm and PDI, and (F) laser diffraction data showing particle size expressed in μm. The data show the efficiency of milling after 1, 3, 6, 12, and 24 h (hours). Data presented as means ± SD or means + SD, *n* = 5.

LD data showed large particles (20–80 μm) after 1 h milling with the three used surfactants and reduced particle sizes less than 1000 nm after 3 h of milling, indicated by d­(*v*) 10 – d­(*v*) 90 values (Figure 2B,D,F). LD data also confirmed that using Tween 80 for 24 h was the most efficient surfactant to produce the smallest N-PCs. To this end, performing LD for the nanosized formulations was necessary as DLS alone could not reliably determine the nanomilling efficiency of the aforementioned experiments, due to the insufficient ability of DLS to determine large particles present within the samples during the initial time points, namely, after 1 and 2 h.

To incorporate the formulations within the MNs and to improve the stability, freeze-drying of the nanosized and micronized PCs was performed. Redispersion of the freeze-dried formulations showed no significant changes in terms of particle size according to DLS and LD (Figure 3A–C). DLS data revealed small changes, indicating a slight agglomeration of N-PCs that was further confirmed by the d­(v) 0.90 LD data (Figure 3A,B).

**Figure 3:**
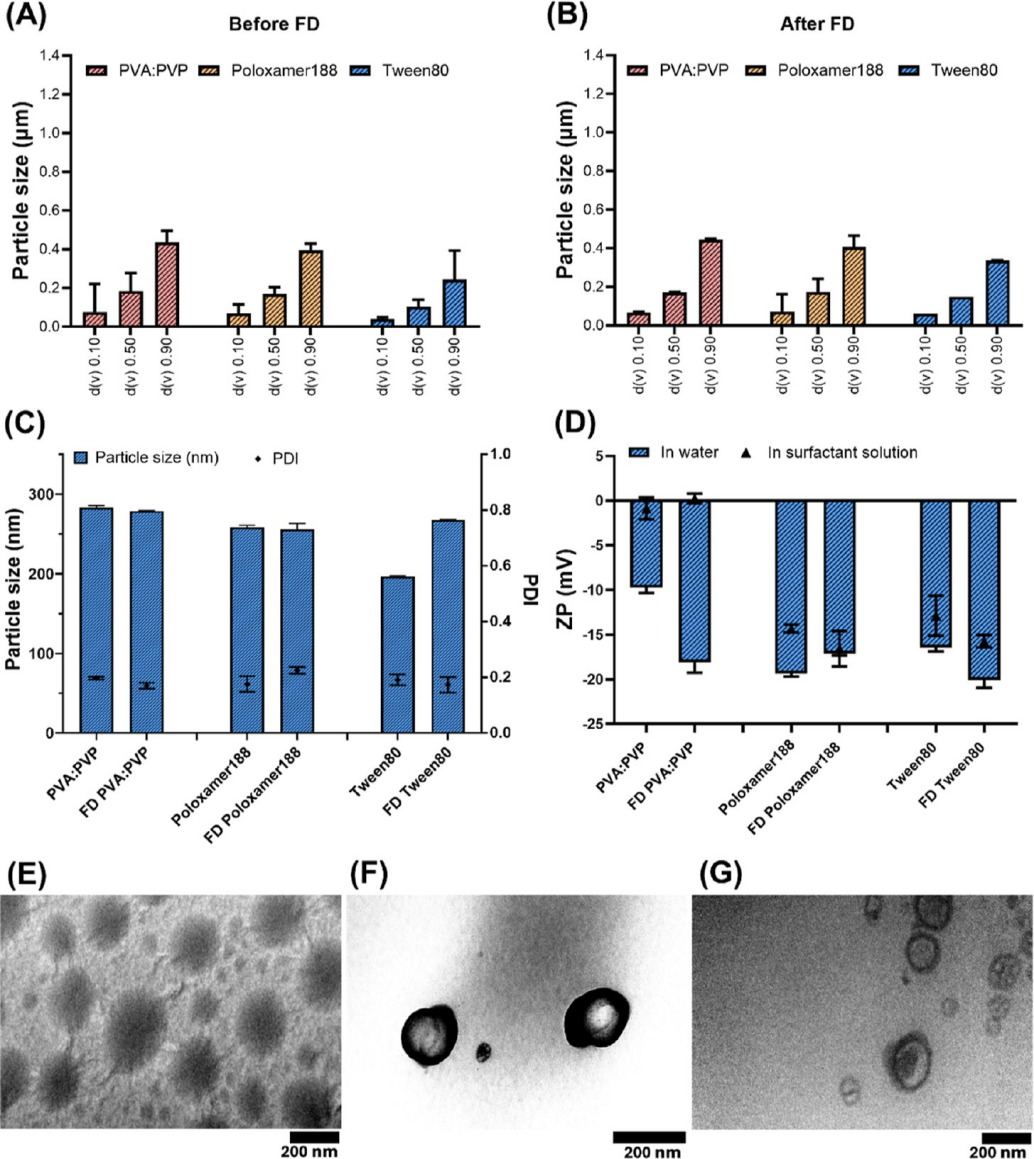
Physicochemical characterization of PCs nanomilled using PVA/PVP (poly­(vinyl alcohol)/poly­(vinylpyrrolidone) K29–32), Poloxamer 188, and Tween 80 as surfactants, showing laser diffraction data showing particle size expressed in μm (A) before FD (freeze-drying) and (B) after FD. (C) DLS data expressed as particle size in nm and PDI. (D) ZP (zeta potential) values measured in water and surfactant solution. TEM images of PCs nanomilled using (E) PVA/PVP (poly­(vinyl alcohol)/poly­(vinylpyrrolidone) K29–32), (F) Poloxamer 188, and (G) Tween 80. Data presented as means ± SD or means + SD, *n* = 5.

The ZP values were also similar for the three screened surfactants when measured in both media (Figure 3). The ZP data indicated no differences of AE N-PCs in the original dispersion media (i.e., surfactant solutions) and the conductivity-adjusted water. On the one hand, analyzing the ZP in the original surfactant solution is believed to accurately reflect the particle charge during storage and is crucial for predicting the real physical stability of the PCs. Furthermore, measuring ZP in water can dilute the PCs and cause the surfactant, which is only loosely bound to the surface, to desorb. To this end, comparing the ZP values in water and the original dispersion medium can determine the strength of the bond between the surfactant and the N-PC surface. A smaller difference indicates a stronger bond and more effective stabilization by the surfactant. As such, this was found with respect to the N-PCs milled with Tween 80, followed by Poloxamer 188, and then PVA/PVP (Figure 3D). The higher ZP values in the original dispersion medium indicated a thicker diffusion layer and greater stability of the N-PCs obtained, which can assist in predicting long-term stability. This interference is more common in dispersed samples containing electrolytes. This was further proved by the TEM images of the N-PCs, as the samples prepared with PVA/PVP exhibited a very thin diffusion layer surrounding the nano-PCs (Figure 3E), which is concordant with the ZP obtained. However, the surrounding diffusion layer of the N-PCs produced with Poloxamer 188 was thicker, followed by Tween 80 (Figure 3F,G). This is consistent with other published studies, which stress the critical role of surfactant selection in achieving stable nanocrystals.^46^

DSC data showed that the endothermic melting peak at 224 °C of AE was present in the three different AE N-PCs prepared in this study (Figure 4A). Furthermore, the formulation prepared with Poloxamer 188 showed a peak at 52 °C that represents the melting point of the used stabilizer. The presence of only one peak of AE in the other two formulations (i.e., with PVA/PVP and Tween 80) could be attributed to the melting point of the used surfactants occurring outside of the analysis range. Furthermore, it can be seen that the melting point attributed to the AE shows a shift toward lower temperatures due to the reduced particle size, which also contributes to the melting point depression as explained by the Gibbs–Thomson equation. This indicates a reduction in the drug crystallinity. Also, the use of certain stabilizers such as Poloxamer 188 and PVA/PVP shows not only a peak shift, but an area reduction, indicating that choice of stabilizer contributes to changes in the crystallinity.

**Figure 4:**
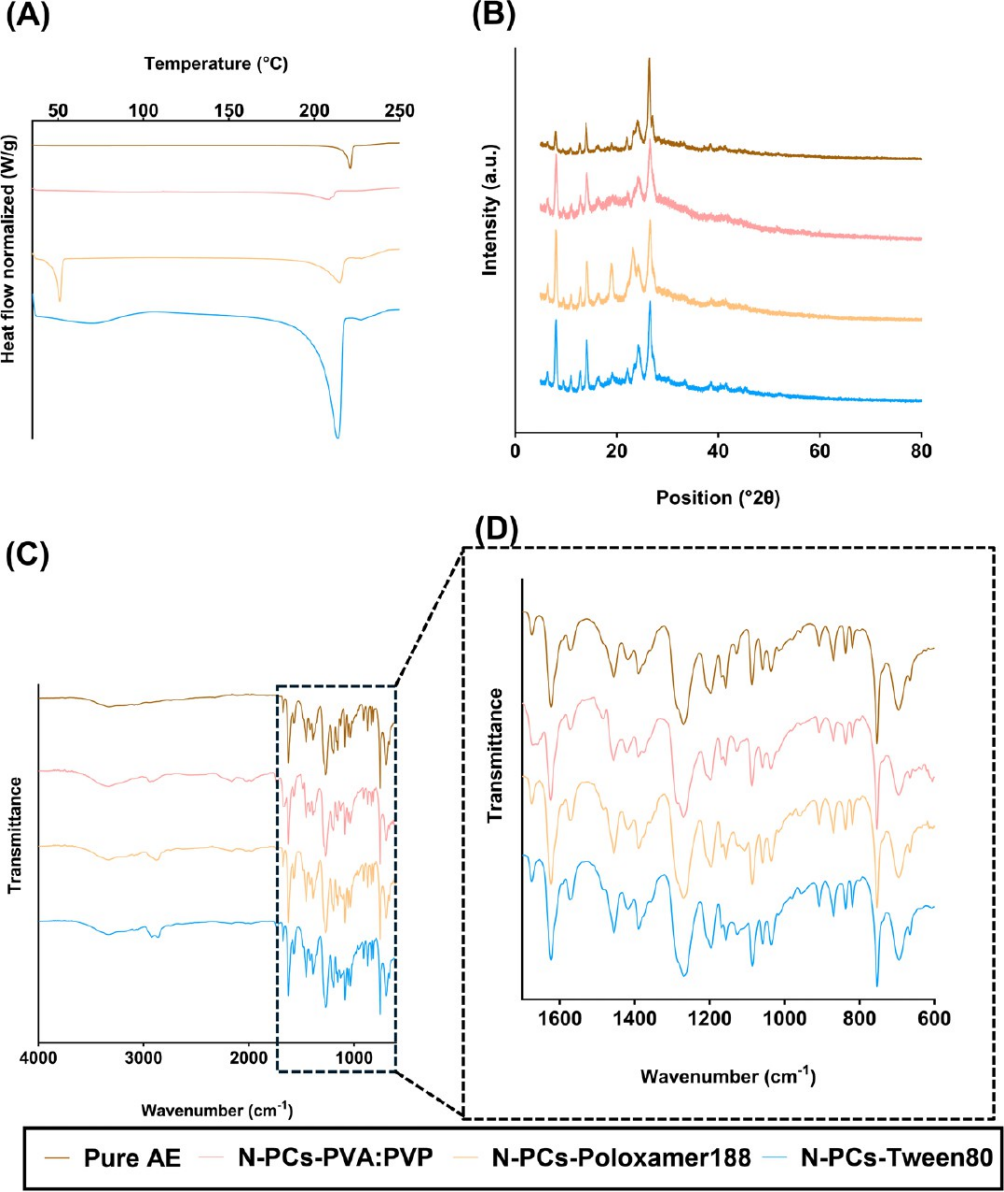
Characterization of the crystallinity/amorphous state of AE (Aloe emodin) N-PCs nanomilled using PVA/PVP (poly­(vinyl alcohol)/poly­(vinylpyrrolidone) K29–32), Poloxamer 188, and Tween 80 as surfactants, showing (A) the DSC thermograms, (B) X-RD diffractograms, and (C) FTIR spectra. (D) FTIR spectra magnified associated with the specific groups.

X-RD analysis was used to compare the fundamental molecular vibrations of AE in the different obtained N-PCs with pure AE. This technique revealed several peaks at diffraction angles between 10.0° and 40.0°, which indicate the crystalline form of pure AE is present (Figure 4B). The formulations retain a certain degree of AE crystallinity, but an amorphous halo can also be observed in all of the formulations containing surfactants. The area of this halo correlates with the DSC results, as PVA/PVP and Poloxamer 188 containing formulations showed higher areas for this amorphous halo in the X-RD graphs.

FTIR analysis highlighted that the functional groups of pure AE were similar to those obtained in the spectra of AE N-PCs (Figure 4C,D) and are similar to the data reported in the literature for AE. Interestingly, the bands associated with the benzene ring skeleton vibration peak from AE (ca. 1650–1450 cm^–1^), the stretching vibration peak of –OH (ca. 3500–2500 cm^–1^), and the stretching vibration peak of the carbonyl group (ca. 1870–1650 cm^–1^) were shifted to a higher wavenumber in the N-PCs, suggesting the involvement of these groups in the potential of hydrogen bounds formation with the used surfactants. The lack of obvious changes in the AE N-PCs spectra indicates no chemical reactions took place during the energy-induced milling process. Similar to these findings, previous studies have demonstrated that surfactant-mediated NC production often leads to a reduction in drug crystallinity.^47^ Furthermore, the observed FTIR shifts indicative of hydrogen bonding align with reports of surfactant–drug interactions in other NC systems.^48^ This validates the results achieved and highlights the common mechanisms involved in NC formation and stabilization.

Based on these data, it could be concluded that employment of surfactants screened did not alter the integrity of the drug molecule and thus, all formulations would be regarded as suitable for future incorporation in MNs. However, as Tween 80 exhibited superior characteristics as the most efficient surfactant with respect to obtaining homogeneous particles of a small size with a stable diffusion layer surrounding the N-PCs, a more detailed comparison between the AE M-PCs and the N-PCs nanomilled with Tween 80 was performed and shown in Figure 5. This comparison shows the efficiency of the nanomilling process applied to reduce the particle size from approximately 40 μm [indicated by d­(*v*) 0.50 (Figure 5A)]. Furthermore, DLS data confirmed that the hydrodynamic particle size was less than 200 nm, which is in line with the LD data obtained (d­(*v*) 0.90). Furthermore, the SEM images indicate the efficient milling method as AE M-PCs showed big irregular agglomeration of unmilled AE particles (Figure 5B,C), while TEM images of the N-PCs present homogeneous circular N-PCs surrounded by a diffusion layer of Tween 80 (cf. Figure 3G). This diffusion layer contributes to the negative ZP values obtained (Figure 5D), as explained in the sections above.

**Figure 5:**
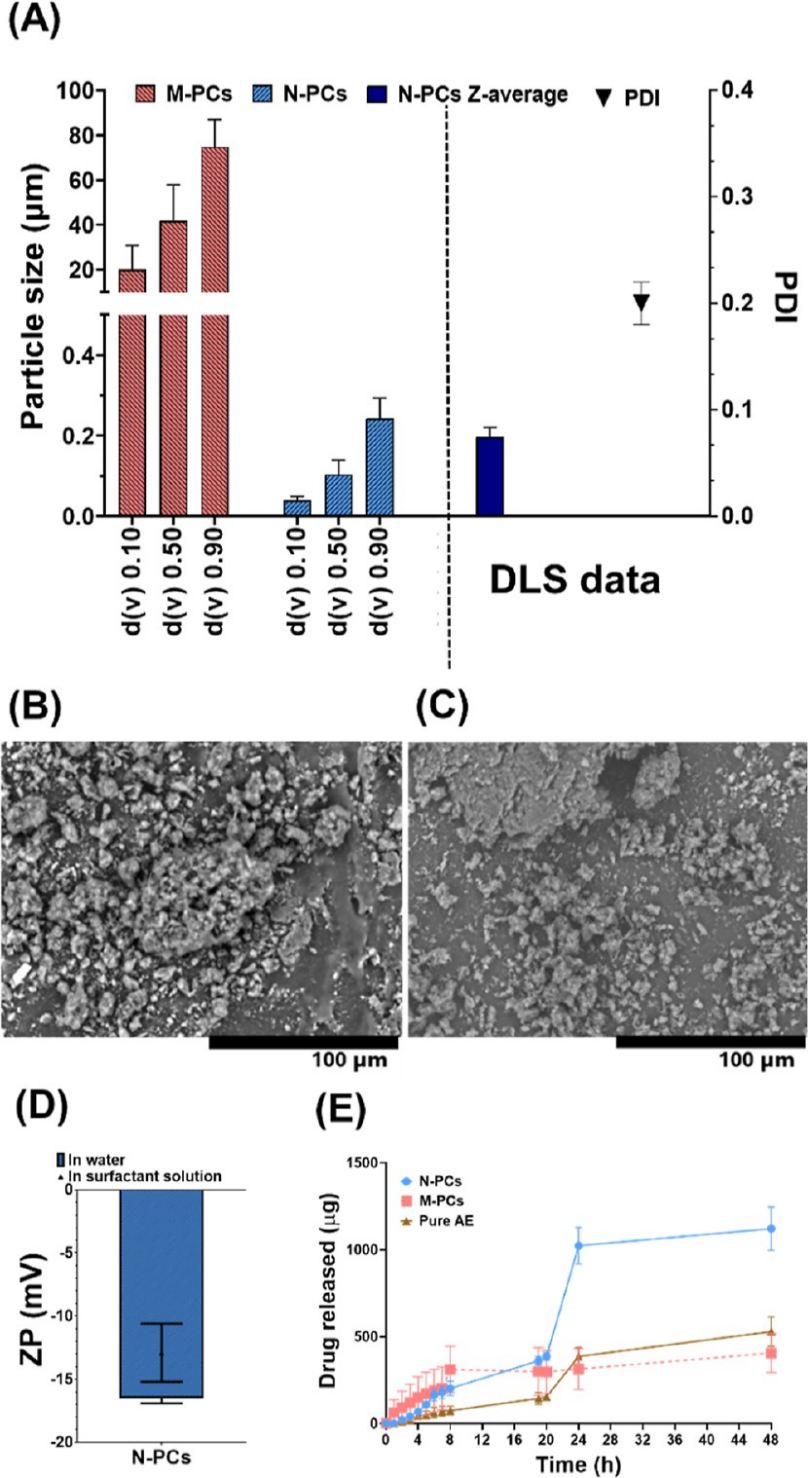
Physicochemical characterization of the chosen N-PCs (nanosized PCs) nanomilled using Tween 80 as surfactants in comparison to the corresponding M-PCs (micronized PCs), showing (A) laser diffraction data showing particle size and DLS data expressed as *z*-average and PDI. SEM images of (B) pure drug, (C) M-PC, and (D) ZP (zeta potential) values of the chosen N-PCs nanomilled using Tween 80 as surfactants measured in water and surfactant solution. (E) The dissolution release profile of the N-PCs and M-PCs nanomilled using Tween 80 as surfactants in comparison to the pure AE (Aloe emodin). Data presented as means ± SD or means + SD, *n* = 5.

Furthermore, the dissolution release profile of the chosen N-PCs milled with Tween 80, compared to AE M-PCs (i.e., unmilled AE) and pure AE (used as bench controls), was studied. For all formulations and the pure AE examined, a biphasic release profile featuring an initial burst of release over the first 4 to 8 h, followed by a consistent, sustained release for up to 48 h, was observed. These findings are in line with published data on nanoformulations, highlighting the established impact of nanosized drug molecules on the kinetic solubility and dissolution rate of poorly soluble compounds.^21^ However, this study also reveals that even small proportions of larger particles in a nanosized formulation can influence the overall release of the active ingredient. Specifically, the 10% of undissolved AE in the N-PCs can be linked to the presence of 10% larger particles in the formulation (Figure 5A), as the d­(*v*)­0.9 value of 243 nm indicates that 90% of the particle volume is equal to or smaller than this specified size (Figure 5A). While these smaller particles were able to dissolve, the larger particles in the AE M-PCs, measuring up to 75 μm, had a lower dissolution rate and kinetic solubility, resulting in the remaining undissolved. To this end, the release profile of AE was well improved after the nanomilling process, reaching more than 1000 μg after 24 h (>60% of the used controls) (Figure 5). The DSC, X-RD, and FTIR provide evidence that the drug molecule retains its crystallinity in both formulations (M-PCs and N-PCs), as explained in Figures 4 and 6. This is in line with the data reported by Udabe et al., depicting that NC formulations enabled more than 80% of the API to be released over an extended period of time, demonstrating that NCs permit improved release profiles for hydrophobic drugs.^49^

**Figure 6:**
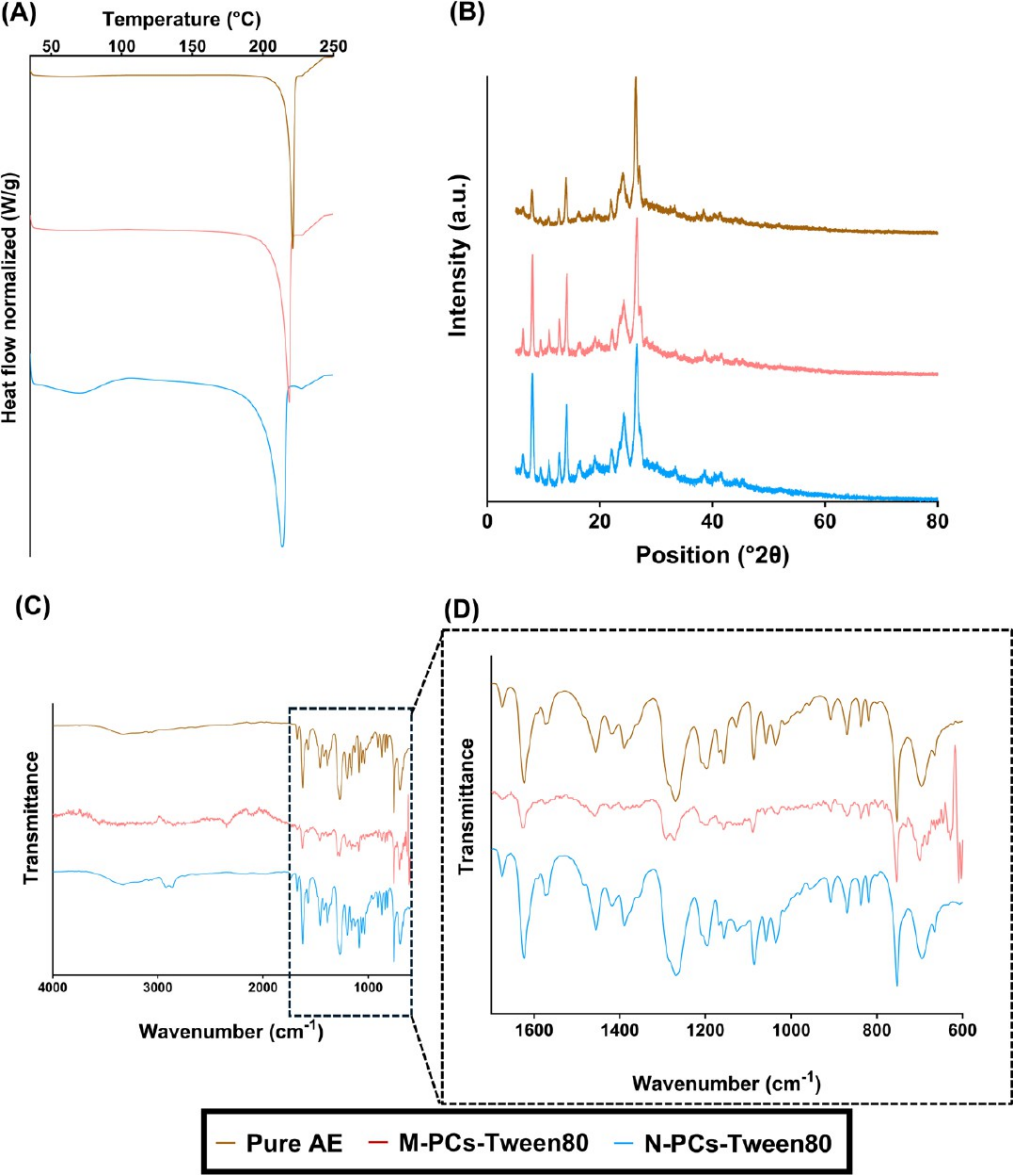
Characterization of the crystallinity/amorphous state of AE (Aloe emodin) N-PCs (nanosized PCs) and M-PCs (micronized PCs) milled using Tween 80 as surfactants and compared to the pure AE showing (A) the DSC thermograms, (B) X-RD diffractograms, and (C) FTIR spectra. (D) FTIR spectra magnified associated with the specific groups.

M-PCs and N-PCs were characterized by using DSC, X-RD, and FTIR (Figure 6). The results obtained are similar to those reported in Figure 4. The DSC curves of the formulated AE showed the melting point of the pure AE but showed a slight shift, due to a reduction in crystallinity. This can be seen in X-RD where the presence of an amorphous halo can be seen, whereby this effect is more obvious in M-PCs. FTIR analysis does not show the presence of new peaks, suggesting that no chemical reactions took place during the milling process. The FTIR of M-PCs showed slight differences when compared to pure AE and N-PCs, as the peak at around 1350 cm^–1^, shows slight shape changes, indicating a different interaction between the surfactant (Tween 80) and AE. These results are consistent with DSC and X-RD, detailing a higher percentage of amorphous AE and thus indicating a higher degree of interactions between AE and the surfactant.

As such, the data confirm the efficiency of the milling process of AE. The milling kinetics can be explained by Avrami’s equation, as reported previously.^50,51^ Briefly, the milling process involves an initial phase of compression and deformation, during which the AE particles undergo changes through fracture and cold welding. As the particles deform, they enter a secondary phase characterized by fatigue failure and fragmentation. The resulting fragments can further reduce in size through cycles of compression-deformation and fracture. When fracture becomes the dominant mechanism, the particles continue to reduce, with the extent of size reduction being influenced by the duration of the milling process (i.e., reaching steady state equilibrium). Based on the data obtained in this study, the use of N-PCs milled with Tween 80 was justified, due to the increased release of AE exhibited over 48 h compared to the unmilled and pure AE samples, and therefore, were deemed appropriate for further in vitro testing. As such, the previously screened PC formulations were not taken further within this work.

#### 3.1.2 In Vitro Skin Permeation Study of AE N-PCs

The transdermal penetration properties of AE N-PCs were examined over a 24 h period and compared with the AE M-PCs as well as the AE alcoholic solution as bench controls. The M-PCs showed no transdermal penetration after 24 h. Yet, the N-PCs showed a significant increase in the penetrated amount of AE (more than 2-fold) when compared with the alcoholic solution used in this study (Figure 7A–H). The deposited amount of AE in the skin layers was higher (approximately 50 μg/cm^3^) in the epidermis and the dermis after the N-PCs application than the amount deposited upon the M-PCs application (<12 μg/cm^3^) (Figure 7). These data agree with the previously reported skin penetration profile of curcumin NCs, showing that the microparticles exhibited less efficient delivery through the skin, when directly compared to NCs.^52^ Furthermore, these data were further demonstrated by the microscopic images of the skin post formulation application, as it was visually apparent that more drug was present in the epidermis layer of the M-PCs and the alcoholic solution, with less in the dermis layer (Figure 7A–F). In contrast, the microscopic images of the N-PC dermis layer indicated a greater accumulation of the drug compared to that of the controls used (Figure 7A–F).

**Figure 7:**
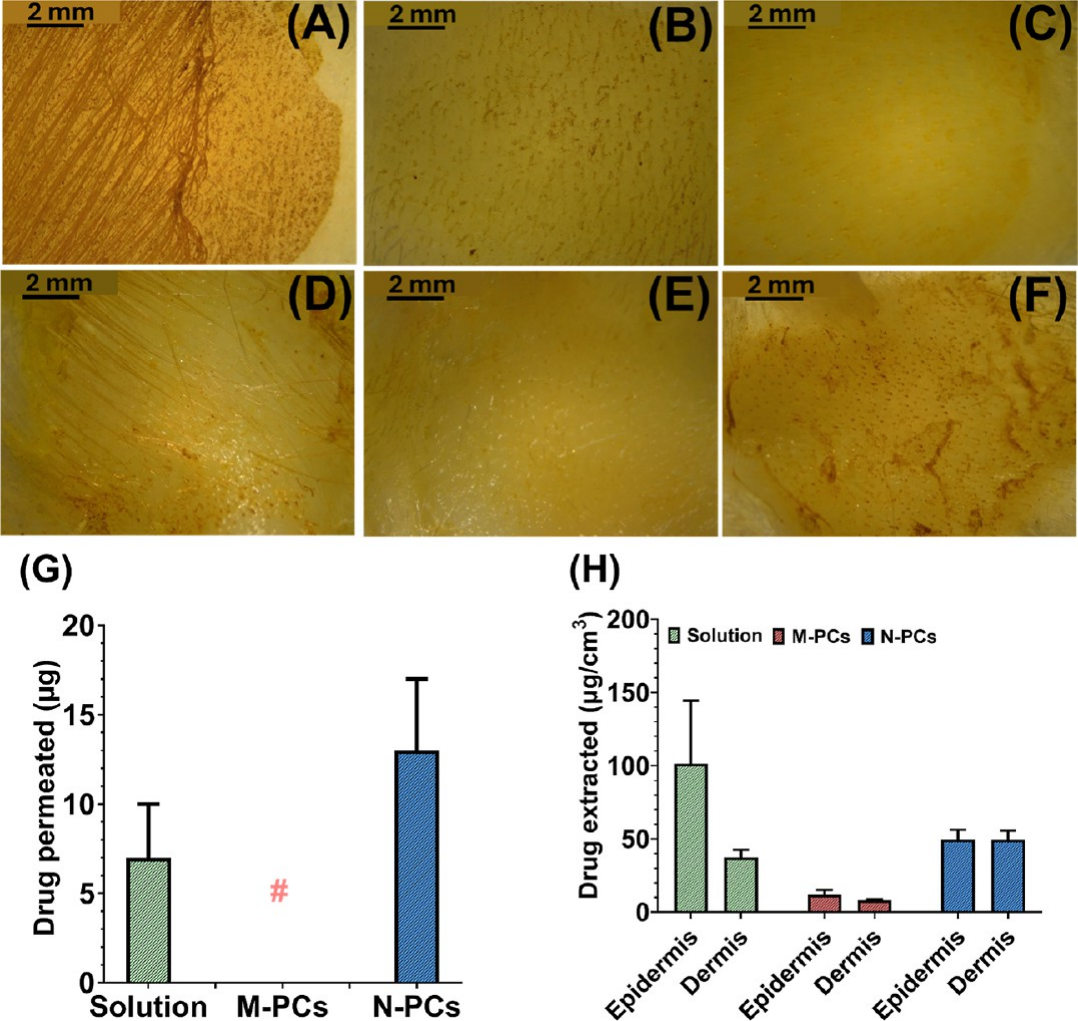
Skin micrographs after application of (A) the solution, (B) M-PCs, and (C) N-PCs. Skin micrographs after removing the epidermis after application of (D) the solution, (E) M-PCs, and (F) N-PCs. (G) Skin permeation profile showing the amount permeated through the skin after the application of the M-PCs and N-PCs. (H) Skin deposition profile showing the amount deposited and extracted from the skin after the application of the M-PCs and N-PCs. # indicates no detectable drug permeation. M-PCs represent micronized PCs, and N-PCs represent nanosized PCs from AE (Aloe emodin). The solution was used as a control. Data presented as means + SD, *n* = 5.

Interestingly, the alcoholic solution used showed a pronounced amount deposited in the epidermis when compared to both micro- and nano formulations used, which could be explained by the possible evaporation, thus building a depot of AE in the SC to be transdermally penetrated, as shown in the penetration profile (Figure 7). This might also be explained by the effects of the materials used on the thickness of the SC as reported previously.^53^ The possible decrease in the thickness of the SC upon the application of an alcoholic solution led to a decreased amount of the drug penetrated but increased the amount deposited on the top layer of the skin (i.e., epidermis). To this end, and despite the improved penetration profile of the N-PCs formulation, in comparison to the M-PCs, a strategy to further improve the deposition/penetration of AE in the skin layers was necessary. To this purpose, the N-PCs and the M-PCs were further incorporated in dissolving MNs, as a novel tool to overcome the restrictive SC, granting access to the deeper layers of the skin, with the potential of an improved deposition of the drug upon dissolution of the needles.

### 3.2 Characterization of AE M-PCs-MNs and N-PCs-MNs

#### 3.2.1 Mechanical Properties and Insertion Profile of the MNs

Dissolving MNs from both M-PCs and N-PCs were produced with low (10% w/w) and high (40% w/w) concentrations of PVA/PVP (1:1). Both the light microscopic images and SEM visualization of the obtained MNs showed a homogeneous structure with a sharp needle tip (Figures 8A–L and 9A–L). MNs obtained with the low PVA/PVP concentration (10%) showed a darker brownish color when compared to the high concentration (40%), which could be attributed to the higher loading capabilities of both AE M-PCs and N-PCs. However, MNs prepared from M-PCs with 10% PVA/PVP showed incomplete tips formation, with missing needles scattered within the array (Figure 8A,B), which can be explained due to the big particle size which hindered the filling of the small tip area, in comparison to that of the small particle size of the PCs obtained by milling, which enabled production of homogeneous MNs with sharp needle tips for both concentrations (Figure 8D–F,J–L).

**Figure 8:**
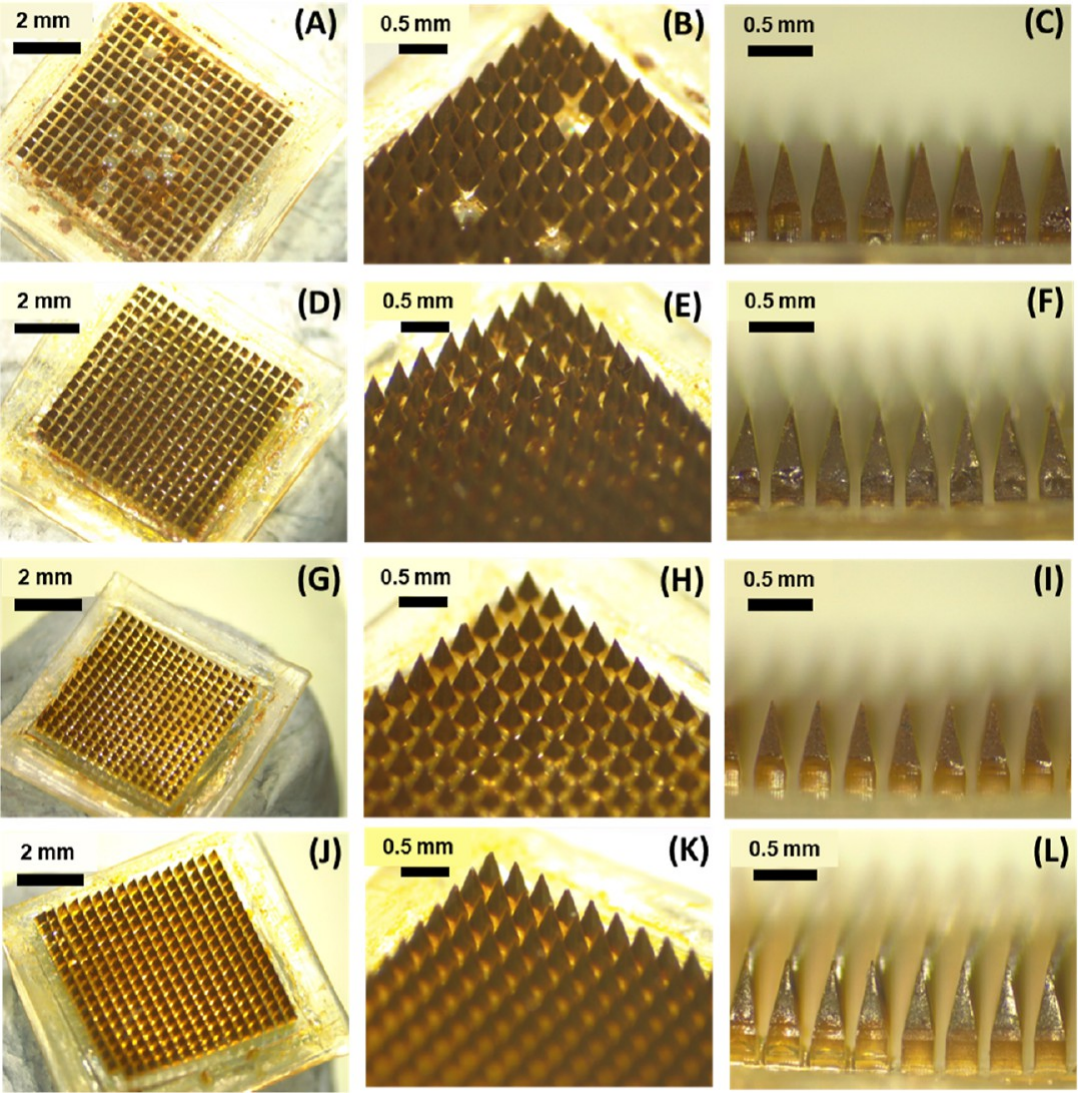
Light microscopic visualization of the obtained MNs in this study. (A–C) MNs obtained by 10% PVA/PVP and containing M-PCs. (D–F) MNs obtained by 10% PVA/PVP and containing N-PCs. (G–I) MNs obtained by 40% PVA/PVP and containing M-PCs. (J–L) MNs obtained by 40% PVA/PVP and containing N-PCs. MNs represent microneedles, M-PCs represent micronized PCs, and N-PCs represent nanosized PCs. PVA/PVP (poly­(vinyl alcohol)/poly­(vinylpyrrolidone) K29–32).

**Figure 9:**
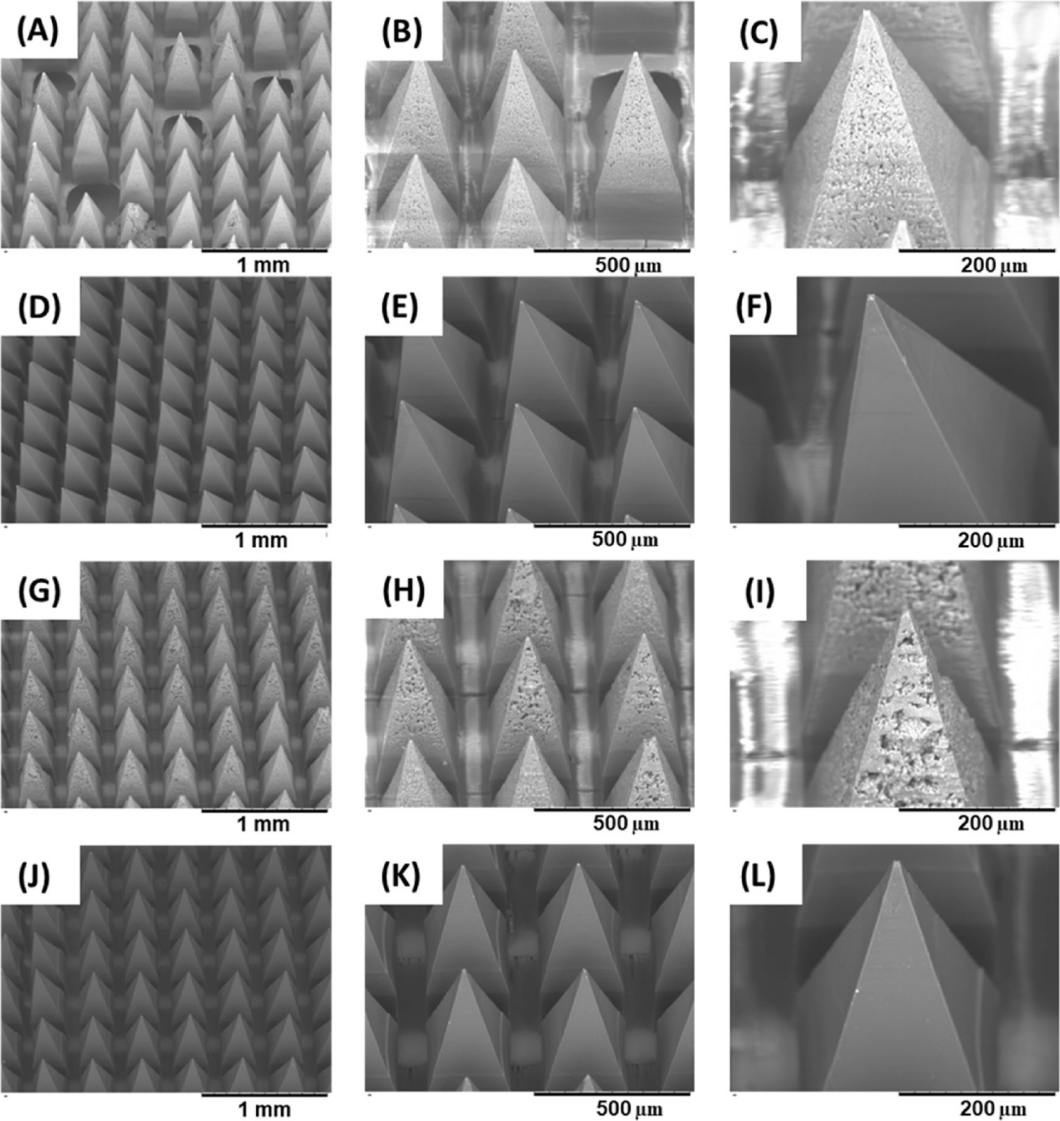
SEM images of the obtained MNs in this study. (A–C) MNs obtained by 10% PVA/PVP and containing M-PCs. (D–F) MNs obtained by 10% PVA/PVP and containing N-PCs. (G–I) MNs obtained by 40% PVA/PVP and containing M-PCs. (J–L) MNs obtained by 40% PVA/PVP and containing N-PCs. MNs represent microneedles, M-PCs represent micronized PCs, and N-PCs represent nanosized PCs. PVA/PVP (poly­(vinyl alcohol)/poly­(vinylpyrrolidone) K29–32).

Furthermore, SEM images of all the M-PCs-MNs showed a porous and nonsmooth microstructure surface of the tips, which reinforces the importance of the particle sizes of the formulations loaded in the MNs. This rough morphology observed could be explained by the large spaces of entrapped air between the microparticles, formed during the drying process, which is not evidenced when fine particles of the nanosized formulations were cast, as demonstrated in Figure 9D–F,J–L. These results indicated that the size of the AE particles encapsulated within the polymeric matrix and the polymeric matrix concentration have an impact on the surface microstructure of the MNs, which agrees with published data of other drugs.^54^

The mechanical strength characteristics of the resultant MNs of both formulations, cast with low and high polymeric matrix concentrations, were analyzed to ensure suitable penetration properties through the SC. A percentage height reduction for each MN formulation was calculated following the application of a 32 N insertion force similar to that exhibited by thumb pressure. The data shown in Figure 10A proved that all the formulations examined indicated a reduction in the needle height of less than 10% and therefore show good mechanical properties, similar to previous work, which demonstrates promising drug delivery in vitro and in vivo.^23,55^ Despite the minor differences between the various formulations, there was no statistical significance exhibited by the lowest and highest height reduction of the N-PCs-MNs at 40% w/w and 10% w/w, respectively (Figure 10A). These results are consistent with the ones reported for MNs prepared with PVA/PVP mixtures and other polymers.^35,55^ Also, during height reduction, no obvious needle fracture was observed. Despite this test not representing the reality of MN application to the skin, as the MNs are applied against an aluminum block, it does serve as a relevant method to compare formulations, ensuring the integrity of the needles and enabling exclusion of potentially brittle formulations. This is crucial not only for application purposes but also to ensure mechanical stability during storage, packaging, and handling. Furthermore, all the MNs produced in this study were tested via insertion into eight layers of Parafilm M, as an accepted model to test the perforation capability of MNs, indicated by the number of holes made upon insertion in each layer.^24^ All the MN formulations could penetrate the fourth layer of Parafilm M (∼500 μm) (Figure 10B), which indicates more than 80% of the needle height penetrated into the artificial membrane. Figure 10B also suggests that increasing the percentage of the polymeric matrix and reducing the particle size of the encapsulated drug could enhance the penetration profile, as shown by the poorer penetration of M-PCs-MNs prepared with 40% PVA/PVP. The insertion profile of these needles does not seem affected by the type of particles loaded. The insertion profile of the MN arrays investigated within this work corresponds to that reported earlier, whereby MN arrays were prepared using the same patch type.^56,57^

**Figure 10:**
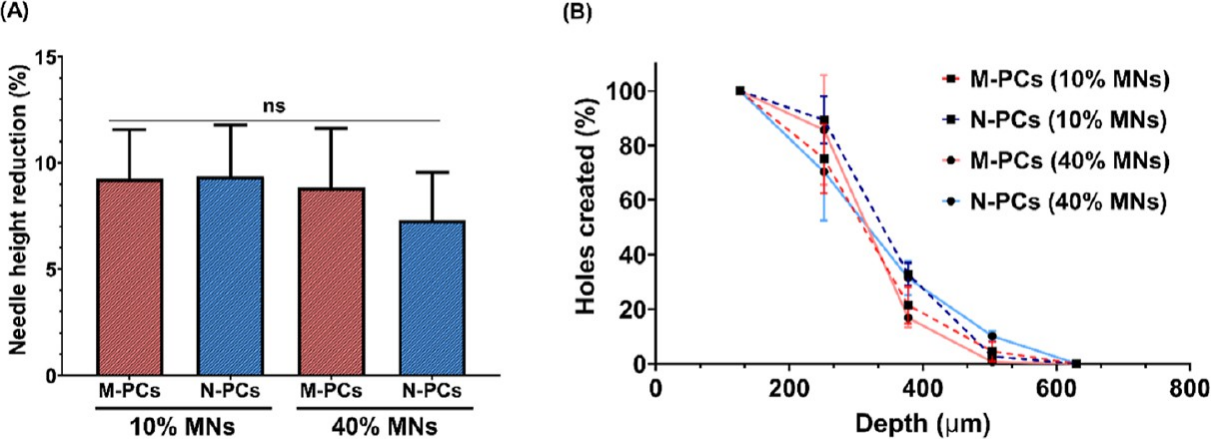
Characterization of the obtained MNs in this study. (A) A comparison of the height reduction percentage of the MNs after the application of a force of 32 N using TA. (B) The percentage of holes created in each Parafilm M layer and the insertion depth after insertion of MNs produced in this study. MNs represent microneedles, M-PCs represent micronized PCs, and N-PCs represent nanosized PCs. The percentage represents the amount of PVA/PVP (poly­(vinyl alcohol):poly­(vinylpyrrolidone) K29–32) used to produce the MNs. Data presented as means ± SD or means + SD, *n* = 4.

To further prove the penetration profile of the obtained MN formulations, a more detailed insertion study was performed on ex vivo neonatal porcine skin, with the insertion profiles determined using OCT imaging (cf. Section 2.2.4.2). As expected, the data showed differences between the artificial membrane and excised porcine skin (Figures 11 and 12).

**Figure 11:**
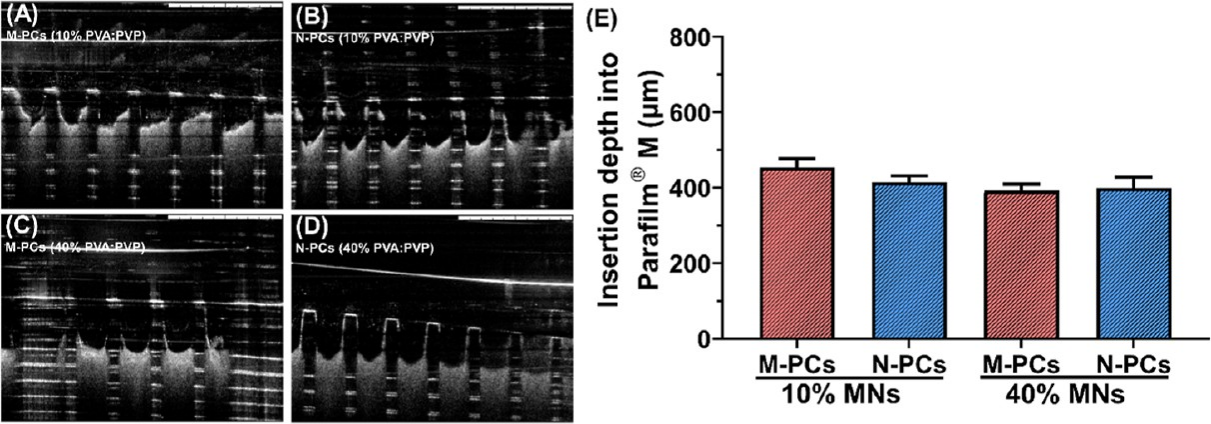
Insertion profile of the MNs obtained in this study in the Parafilm M artificial skin model. (A–D) OCT images of the MNs showing the insertion depth (the scale bar represents a length of 1 mm). (E) Insertion depth analyzed based on the obtained OCT images. MNs represent microneedles, M-PCs represent micronized PCs, and N-PCs represent nanosized PCs. The percentage represents the amount of PVA/PVP (poly­(vinyl alcohol):poly­(vinylpyrrolidone) K29–32) used to produce the MNs. Data presented as means + SD, *n* = 4.

**Figure 12:**
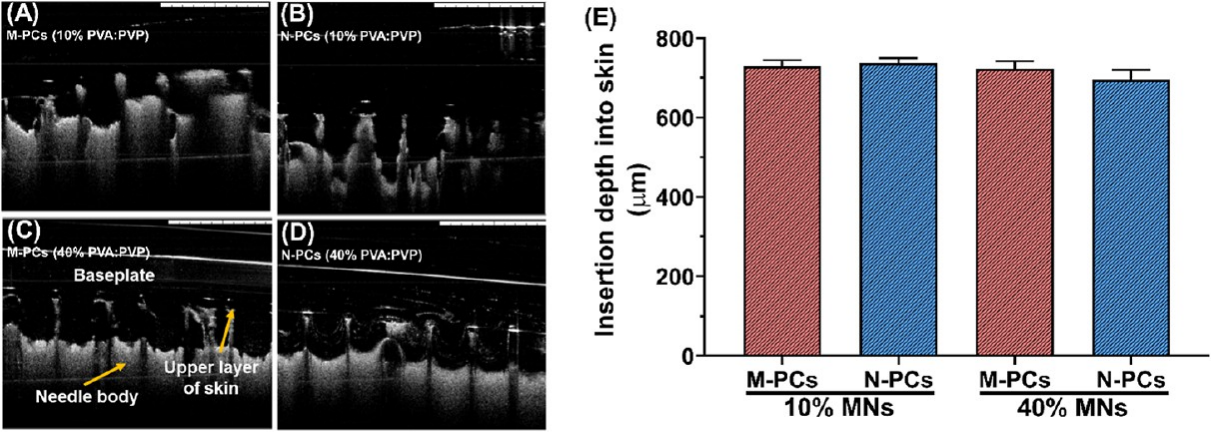
Insertion profile of the MNs obtained in this study in the neonatal porcine skin. (A–D) OCT images of the MNs showing the insertion depth (the scale bar represents a length of 1 mm). (E) Insertion depth analyzed based on the obtained OCT images. MNs represent microneedles, M-PCs represent micronized PCs, and N-PCs represent nanosized PCs. The percentage represents the amount of PVA/PVP (poly­(vinyl alcohol)/poly­(vinylpyrrolidone) K29–32) used to produce the MNs. Data presented as means + SD, *n* = 4.

However, no differences were noticed between the M-PCs-MNs and the N-PCs-MNs fabricated with 10% w/w or 40% w/w polymer blends within each respective subgroup of insertion into either the artificial membrane or excised porcine skin. By contrast, the penetration depth of all the formulations was significantly affected, leading to approximately 2-fold deeper insertion in the skin when compared to the artificial model (Figure 12). These differences could be explained by the different mechanical properties of both systems and the hydration of the real skin as described previously.^24,58^ Furthermore, this can be explained by the presence of the polymeric matrix (PVA/PVP) of the MNs and the surfactant (Tween 80) in the AE formulations that were encapsulated in the MNs. The polymeric matrix and Tween 80 as wetting agents can reduce the surface tension at the solid–liquid interface, thus facilitating the spreading and penetration of the liquid (i.e., skin interstitial fluid (ISF)) of the full-thickness porcine skin into the solid hydrophilic polymeric matrix. The ISF wets the surface of the MNs, which in turn decreases the friction between the two surfaces (i.e., the real skin and the MNs), promoting a well-improved insertion profile in the skin, compared to that of the artificial model.^24^ To this end, all of the MNs obtained are deemed strong enough to be inserted in the skin and thus appropriate for in vitro testing.

#### 3.2.2 Drug Content within the MNs

Following the analysis of the mechanical properties and insertion profile of AE PCs-MNs, the encapsulated AE content was determined for all of the MNs produced. The data demonstrated a higher amount loaded within the MNs made of 10% w/w polymeric matrix, with a content of approximately 0.28 mg and 0.45 mg for M-PCs-MNs and N-PCs-MNs, per microarray patch (Figure 13), respectively. By contrast, increasing the amount of polymer mixed with AE led to a lower loading efficiency (Figure 13). Interestingly, no significant difference in the AE-loaded amount was noticed between M-PCs-MNs and of N-PCs-MNs made with the 40% polymeric matrix.

**Figure 13:**
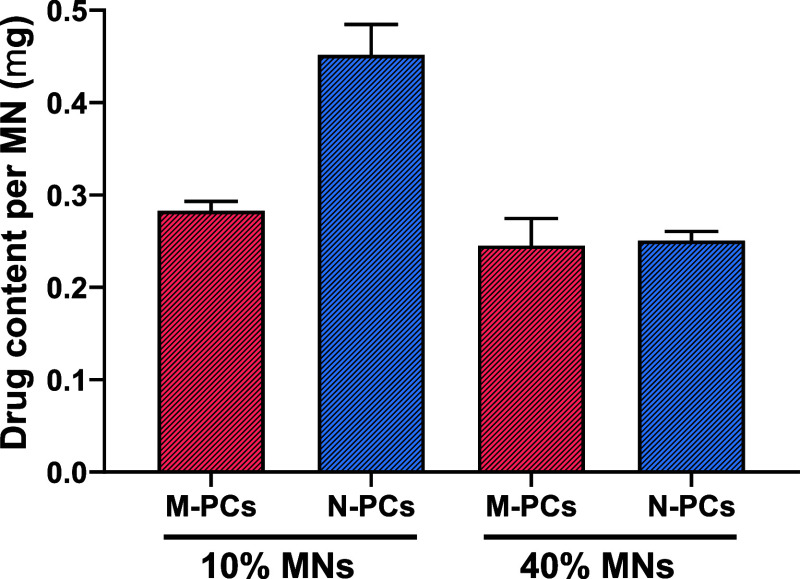
Drug content within the MNs obtained in this study. MN represents microneedles (i.e., microneedle array patch), M-PCs represent micronized PCs, and N-PCs represent nanosized PCs. The percentage represents the amount of PVA/PVP (poly­(vinyl alcohol)/poly­(vinylpyrrolidone) K29–32) used to produce the MNs. Data presented as means + SD, *n* = 4.

#### 3.2.3 In Situ Skin Dissolution Studies of the MNs

The in situ dissolution test was explored as an important test to determine the time required for the dissolving MN formulation to be solubilized after application. Figure 14 shows the different dissolution profiles of the four formulations obtained in this study. The M-PCs-MNs did not show a complete dissolution profile with parts of the tip remaining after application within the skin for 60 min, as indicated in the microscopic images (Figure 14). This was demonstrated in both concentrations of the polymeric matrix (i.e., 10% and 40%). Despite this, the N-PCs-MNs showed an improved in situ dissolution profile even after 30 min of application (Figure 14C,G), with an even faster dissolution profile exhibited by the increased polymeric matrix concentration of 40% (Figure 14G,H). This might be attributed to the large particle size of the M-PCs hindering the wetting occurring in situ, as the smaller surface of nanosized particles, when compared to the micronized particles, permits faster wetting. Furthermore, the higher the polymeric concentration of the hydrophilic matrix, the faster the wettability and thus, the quicker dissolution could be achieved. As such, it may be concluded that the formulation showing the most promising characteristics is the N-PCs-MNs made from a 40% PVA/PVP matrix. In addition to the better wettability of nanosized formulations in comparison to the microcrystals, the synergistic effect of PVA/PVP and Tween 80 promotes the PCs’ wettability and solubility, and therefore, the dissolution of the MNs. These data are in line with a previous work reported by Chowdary and Rao and Brough et al., showing that the efficiency of such a combination improves the wettability, leading to faster dissolution and solubilization.^59,60^

**Figure 14:**
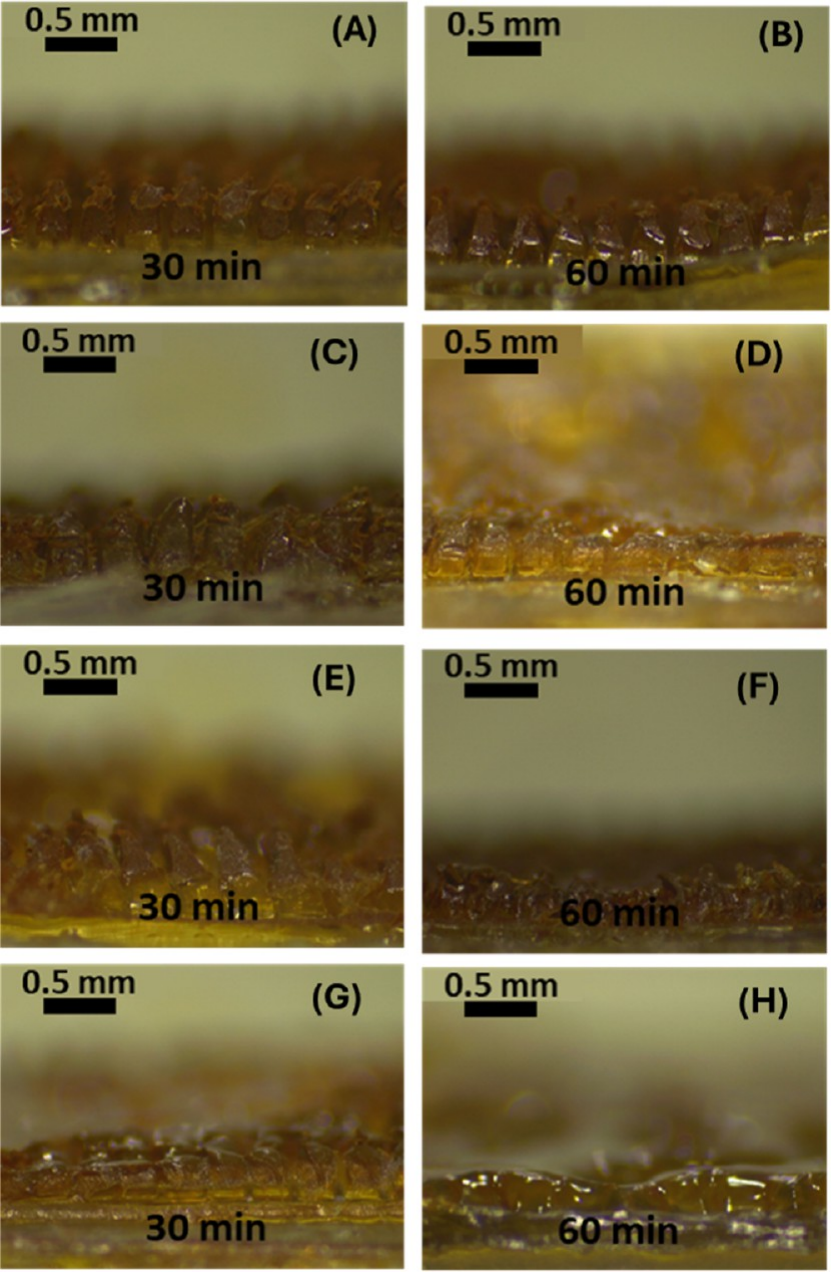
In situ dissolution study showing the MNs dissolved after 30 and 60 min of insertion into the skin. (A,B) MNs obtained by 10% PVA/PVP and containing M-PCs. (C,D) MNs obtained by 10% PVA/PVP and containing N-PCs. (E,F) MNs obtained by 40% PVA/PVP and containing M-PCs. (G,H) MNs obtained by 40% PVA/PVP and containing N-PCs. MNs represent microneedles, M-PCs represent micronized PCs, and N-PCs represent nanosized PCs. The percentage represents the amount of PVA/PVP (poly­(vinyl alcohol)/poly­(vinylpyrrolidone) K29–32) used to produce the MNs.

This dissolution profile was also confirmed with microscopic images of the skin after removing the MNs. AE could be deposited in the skin as indicated by the brown color (Figure 15). The formulation containing N-PCs-MNs with needles composed of a 40% polymeric matrix showed the greatest efficiency, allowing fast deposition of the photosensitizer in the skin visible after 30 min. To this end, it could be concluded that the polymeric matrix and the particle size of the formulation not only affect the characteristics of the MNs but also impact the dissolution profile of the dissolving MNs. Based on this, the dissolving MNs manufactured with the higher concentration (i.e., 40%) of the polymeric matrix were chosen for the in vitro testing.

**Figure 15:**
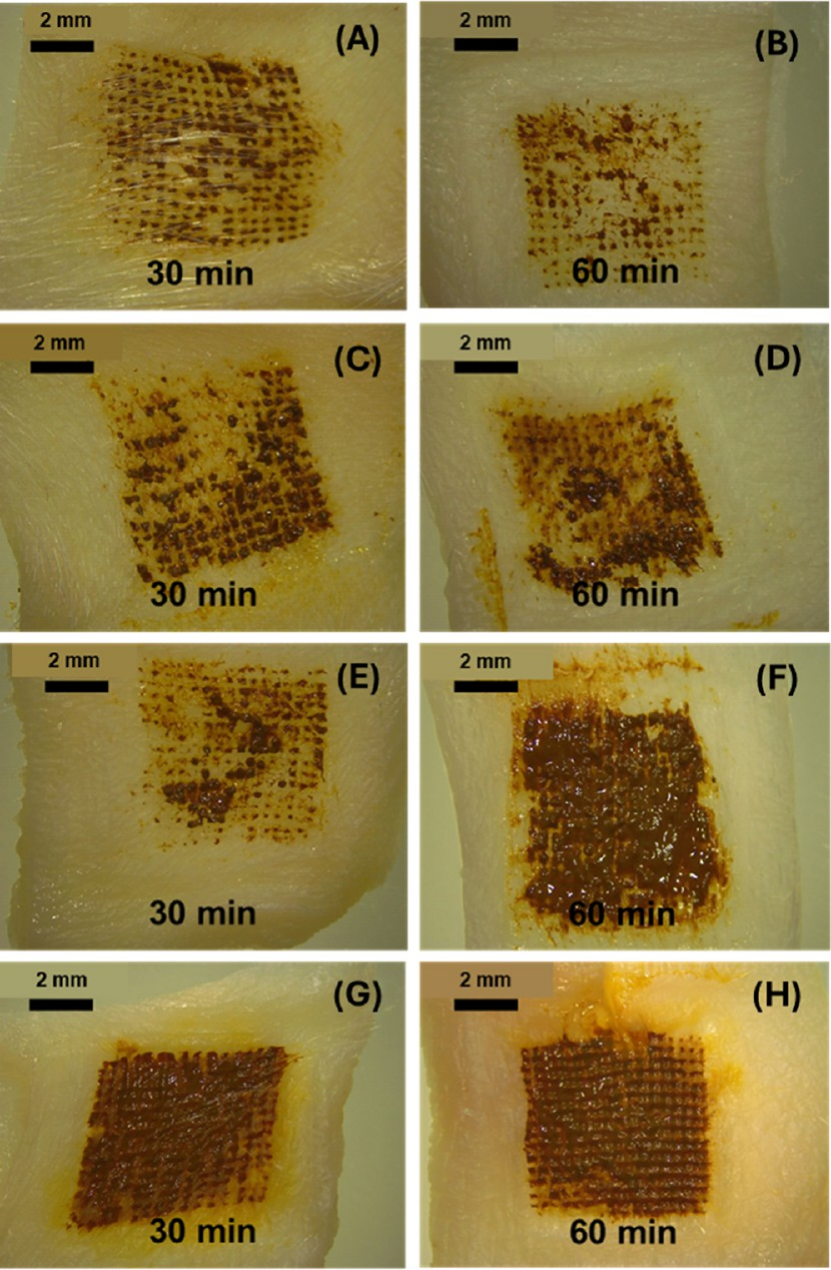
Skin microscopic images taken for the in situ dissolution study showing the MNs dissolved after 30 and 60 min of insertion within the skin. The first panel shows the skin after applying MNs obtained by 10% PVA/PVP and containing M-PCs, presenting (A) skin after MNs insertions and dissolving into the skin (30 min), and (B) skin after MNs insertions and dissolving into the skin (60 min). The second panel shows the skin after applying MNs obtained by 10% PVA/PVP and containing N-PCs, presenting (C) skin after MNs insertions and dissolving into the skin (30 min), and (D) skin after MNs insertions and dissolving into the skin (60 min). The third panel shows the skin after applying MNs obtained by 40% PVA/PVP and containing M-PCs, presenting (E) skin after MNs insertions and dissolving into the skin (30 min), and (F) skin after MNs insertions and dissolving into the skin (60 min). The fourth panel shows the skin after applying MNs obtained by 40% PVA/PVP and containing N-PCs, presenting (G) skin after MNs insertions and dissolving into the skin (30 min) and (H) skin after MNs insertions and dissolving into the skin (60 min). MNs represent microneedles, M-PCs represent micronized PCs, and N-PCs represent nanosized PCs. The percentage represents the amount of PVA/PVP (poly­(vinyl alcohol)/poly­(vinylpyrrolidone) K29–32) used to produce the MNs.

#### 3.2.4 Physicochemical Characterization of AE Formulations within the Selected MNs

The physicochemical properties of the AE M-PCs and N-PCs were characterized after dissolving the MNs. This is an essential step to confirm that the incorporation of the nanosized and micronized AE within the MNs has no significant impact on the particles and does not change their properties, with regard to size, homogeneity of the particles, and surface potential. The obtained data show that the particle size of the nano and micro formulations was doubled after formulation into the MNs and subsequent dissolution of the polymer. However, it was determined that this may be due to agglomerates formed after mixing with the polymers, the casting process, or absorbance of the polymers around the drug particles. This was proved by washing the particles and measuring the subsequent particle size. As such, the particle size of the PCs after washing is about 250 nm, as shown in the DLS data (Figure 16), which is similar to the original PCs before incorporating them in the MN formulation. Furthermore, to further highlight the absence of agglomerates or any remaining large particles, LD measurements were performed, and the results indicated that the particles observed were similar to the original formulation, highlighting that there are no effects of incorporating the nanosized AE within the MNs.

**Figure 16:**
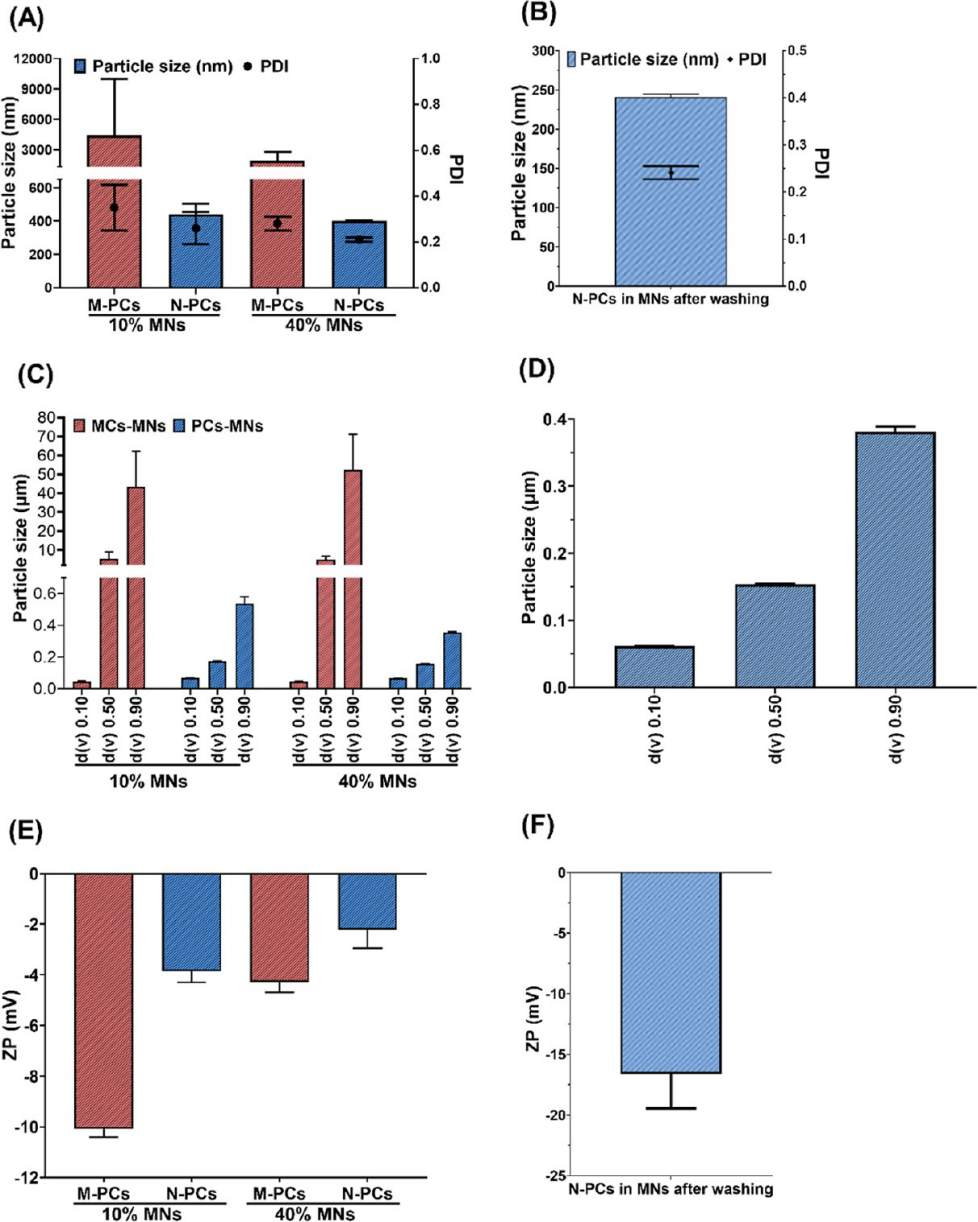
Physicochemical characterization of the N-PCs and M-PCs incorporated with the MNs before washing and the chosen formulation N-PCs made from 40% PVA/PVP MNs after washing. (A) DLS data expressed as *z*-average and PDI before washing and (B) DLS data expressed as *z*-average and PDI after washing of N-PCs made from 40% PVA/PVP MNs. (C) Laser diffraction data showing particle size before washing and (D) laser diffraction data showing particle size after washing of N-PCs made from 40% PVA/PVP MNs. (E) ZP (zeta potential) values before washing and (F) ZP (zeta potential) values after washing of N-PCs made from 40% PVA/PVP MNs. MNs represent microneedles, M-PCs represent micronized PCs, and N-PCs represent nanosized PCs. The percentage represents the amount of PVA/PVP (poly­(vinyl alcohol)/poly­(vinylpyrrolidone) K29–32) used to produce the MNs. Data presented as means ± SD or means + SD, *n* = 5.

The ZP values also showed variation after dissolving the MNs as a result of the layer of the polymers attached to the particles, which led to a significant shift to approximately −4 mV. This might be explained by PVP’s known ability to form complexes with APIs and PVA’s extensive number of OH groups, which can interact with the API cargo forming H-bonds, as previously described by Newman and Zografi.^61^ Similar to the particle size and PDI values, after washing cycles, the ZP value was comparable to the original PCs formulation.

DSC, X-RD, and FTIR of the N-PCs-MNs were also performed to ensure that the drug remained intact during the fabrication process; to permit AE particles release upon MN dissolution. The data showed no changes were detected in AE properties as the data provided similar thermogram, diffractogram, and peaks for DSC, X-RD, and FTIR, respectively, in comparison to the pure AE and physical mixture (Figure 17).

**Figure 17:**
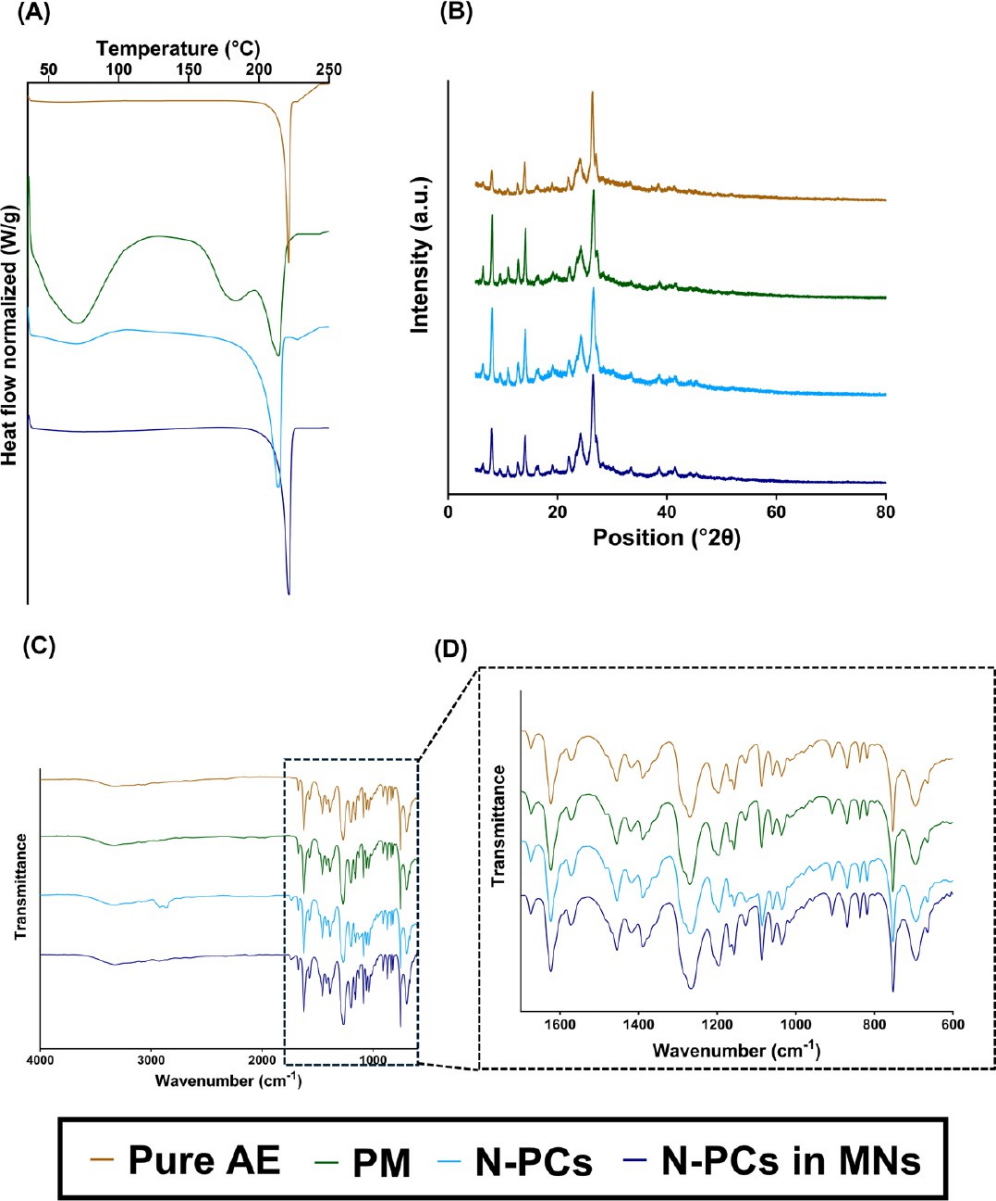
Characterization of the crystallinity/amorphous state of chosen N-PCs (nanosized PCs) after incorporating them within the MNs and compared to N-PCs before incorporating them within the MNs, the pure AE and the corresponding PM (physical mixture) showing (A) the DSC thermograms, (B) X-RD diffractograms and (C) FTIR spectra. (D) FTIR spectra magnified associated with the specific groups. MNs represent microneedles, and N-PCs represent nanosized PCs.

#### 3.2.5 Stability Study of the Selected MN Formulations

Accelerated and long-term stability studies for nano- and micro-AE MN formulations resulted in less than a 20% height reduction, which is similar to the data obtained for the freshly prepared MNs. Furthermore, this value is considered acceptable and concordant with previous studies.^35,55^ To this end, it could be concluded that all the AE MNs prepared in this study demonstrated good mechanical stability. To further prove that AE was also stable during the accelerated and long-term stability studies, the drug content was analyzed. The drug contents obtained were similar for all the formulations examined in the stability study, but the M-PCs formulation demonstrated a slight variation of the drug contained within the MNs (Figure 18). This could be credited to the inclusion of bigger particles, in comparison to the nanosized PCs which can fill MN mold in a more homogeneous manner. Ultimately, it could be concluded that the produced N-PCs MNs were stable, and the drug loaded was intact and remained stable during the period of study, which indicates that the drug is still effective upon dissolution and release from the polymeric matrix of the MNs. The MN polymeric matrix did not affect the drug properties but provides further protection from the possible degradation, as demonstrated previously by Di Natale et al. and Wang et al.^62,63^

**Figure 18:**
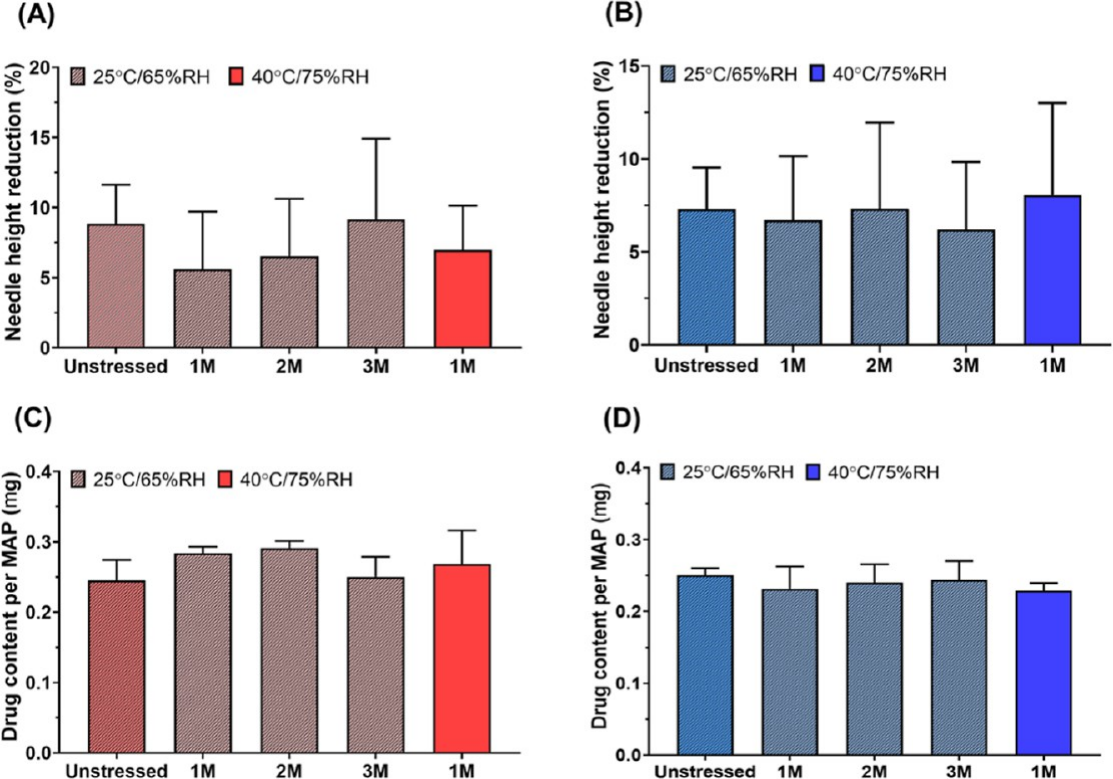
Stability study of the final 40% PVA/PVP MNs showing needle height reduction of MNs containing (A) M-PCs and (B) N-PCs stored at 25 °C/65%RH and 40 °C/75%RH and drug content of MNs containing (C) M-PCs and (D) N-PCs. MAP represents microneedle array patch (i.e., MNs), M-PCs represent micronized PCs, and N-PCs represent nanosized PCs. The percentage represents the amount of PVA/PVP (poly­(vinyl alcohol)/poly­(vinylpyrrolidone) K29–32) used to produce the MNs. Unstressed means MNs tested on day (0) after production, M represents month, and RH is relative humidity. Data presented as means + SD, *n* = 5.

Therefore, the MN formulations investigated here were deemed to be suitable for use in skin studies and antimicrobial PACT, as shown in the following sections.

#### 3.2.6 In Vitro Skin Permeation and Deposition Study of the Selected MNs Formulations

The skin images taken after conducting the in vitro permeation study showed that the M-PCs released from the MNs demonstrated a greater amount of AE in the epidermis layer than the dermis layer, when compared to the N-PCs (Figure 19). In addition, a significantly higher amount of AE from the N-PCs-MNs was transdermally permeated when compared with the M-PCs-MNs. After 24 h, more than 5 μg permeated the nanosized PCs-MNs, but less than 1 μg permeated from the M-PCs-MNs. By contrast, the skin deposition profile showed similar amounts of AE deposited within the skin layers from the MNs of both formulations (∼200 μg in the skin layers) (Figure 19), which are proven to permit further AE release, as expected based on Fick’s law.^5^ Importantly, the deposited amount of both nano- and micro sized formulations was considered sufficient to target the dermal infections in situ without damaging the surrounding tissues or leading to AMR. This is especially poignant due to the fact that AE is effective upon irradiation, allowing a more targeted approach to prove to be advantageous in treating skin infection localized to specific areas. To further prove or disprove this approach, an antimicrobial PACT was tested, as shown in the following sections.

**Figure 19:**
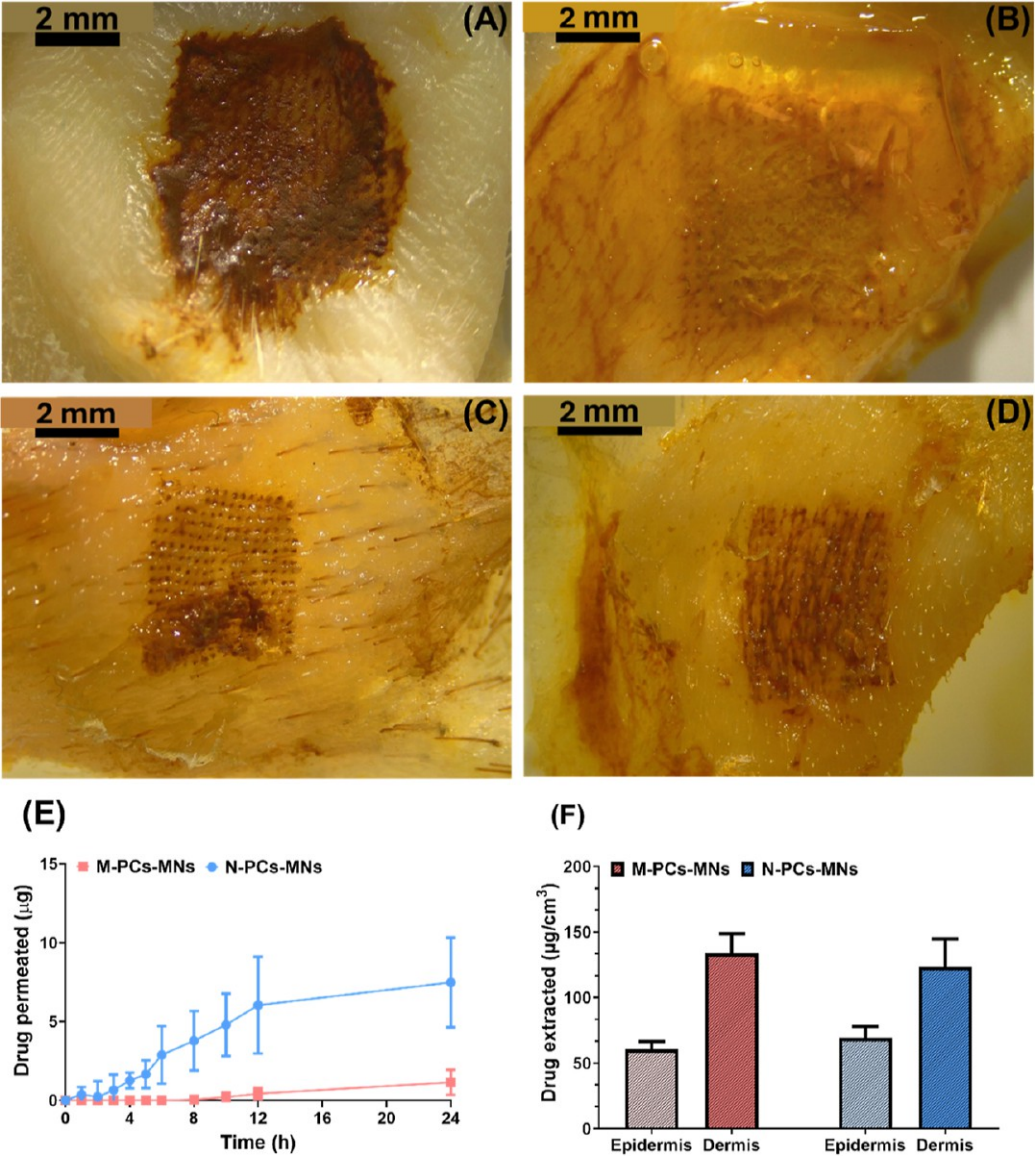
Skin micrographs after application of (A) M-PCs-MNs and (B) N-PCs-MNs. Skin micrographs after removing the epidermis after application of (C) M-PCs-MNs and (D) N-PCs-MNs. (E) Skin permeation profile showing the amount permeated through the skin after the application of the M-PCs-MNs and N-PCs-MNs. (F) Skin deposition profile showing the amount deposited and extracted from the skin after the application of the M-PCs-MNs and N-PCs-MNs. MNs represent microneedles, M-PCs represent micronized PCs, and N-PCs represent nanosized PCs from AE (Aloe emodin). Data presented as means + SD, *n* = 6.

### 3.3 Antimicrobial photodynamic Activity of the Selected Formulations

The chosen AE micro- and nano-PCs were tested regarding their antimicrobial PACT against the Gram-positive *S. saprophyticus*, before and after incorporation into MNs, in both dark and irradiated conditions. The data obtained showed that the micro- and nano-PCs showed no PACT properties under dark conditions (Figure 20). A blank (i.e., the surfactant solution) was employed as a bench control and did not show changes in the activity upon irradiation. However, the antimicrobial properties of the PC formulations were significantly improved upon irradiation, leading to >3.8 log reduction in bacterial viability at their most effective concentrations. The N-PCs showed a 4-fold more pronounced PACT after irradiation compared to the M-PCs (Figure 20). To place each formulation into perspective, a 4 μg/mL concentration of the nanosized PCs was considered similar in terms of efficacy to 16 μg/mL of micronized PCs. Therefore, it can be assumed that PACT from the irradiated PCs showed better activity after nanosization. This can be further explained by the efficiency of the nanomilling, which provides improved solubility of AE and, as a result, enhanced PACT. The data indicated a significantly stronger PACT effect when we utilized a concentration approximately eight times lower than that used by Otieno et al. in their study on Gram-positive bacteria.^64^ While they employed a concentration of 32 μg/mL of AE, our study demonstrated an effective concentration of 4 μg/mL.

**Figure 20:**
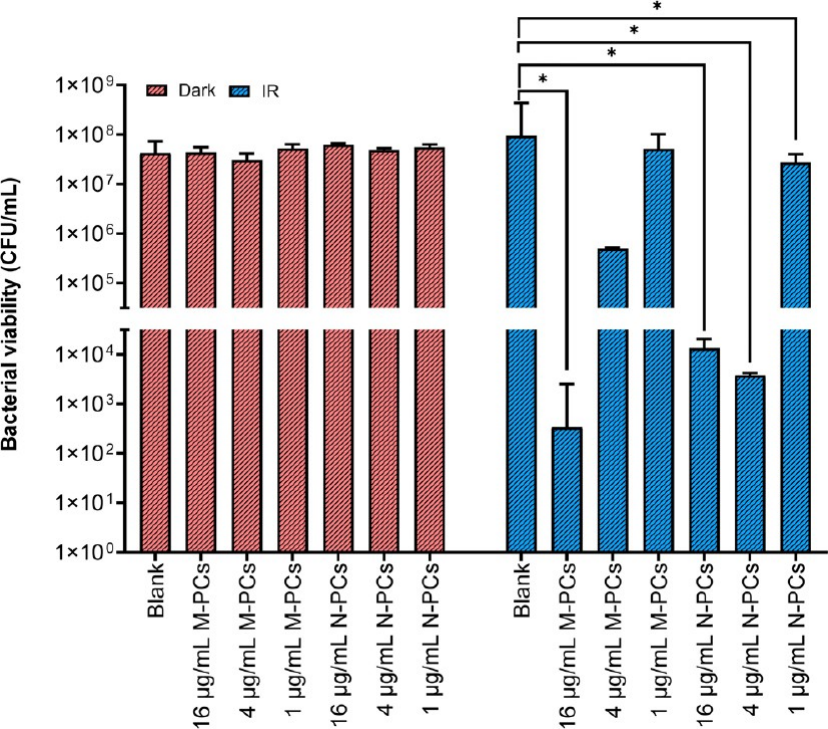
Bacterial viability of *S. saprophyticus* subsp. *bovis* on the obtained M-PCs and N-PCs before (dark) and after (IR) irradiation. Blank formulation (i.e., without drug) was used as a control. M-PCs represent micronized PCs, and N-PCs represent nanosized PCs from AE (Aloe emodin). Data presented as means + SD, *n* = 4.

Treating the Gram-negative bacteria *E. coli* with the AE PhotoCrystals did not affect the bacterial viability (data not shown), which can be attributed to the lower PACT activity of PSs against the Gram-negative bacteria, as demonstrated by previously published results.^65^ The interaction of AE PCs with Gram-negative bacteria may be diminished due to their distinctive cellular structure, e.g., differences in cell wall composition between Gram-negative and Gram-positive bacteria. Furthermore, two main factors restrict the uptake of AE in Gram-negative bacteria. First, the lipid-dense membrane bilayer comprising the cell wall hinders the penetration of AE PCs into these bacteria. Second, porins and efflux pumps that control the intake and expulsion of nutrients and toxins function as inherent resistance mechanisms. However, the concentration of AE used in this study is nearly 10 times lower compared to previous PACT studies by Comini et al., which indicated that AE did not show dark toxicity toward *E. coli* at concentrations of up to 320 μg/mL, but antibacterial effects from irradiated AE were observed at levels exceeding 250 μg/mL.^66^ Therefore, the activity of the developed matrix-free system described here was not further tested with regard to the PACT against *E. coli*.

Importantly, the PACT results stated for the Gram-positive bacteria highlight the effectiveness of the matrix-free PCs system at a considerably reduced AE concentration of just 4 μg/mL. Ultimately, the obtained AE PCs not only exhibited enhanced solubility but also heightened antibacterial properties, facilitating their potential use in skin-targeted therapeutic applications, such as difficult-to-treat dermal infections, when MNs are combined with PACT. To this end, a targeted antibacterial strategy could be demonstrated, using the developed matrix-free micronized and nanosized PCs, but applying the used formulations directly to the skin showed a low amount deposited within the skin layers showing <50 μg and <12 μg for the nanosized and micronized PCs, respectively (cf. Figure 7), which may hinder the efficiency in targeting the deeper skin infections. Therefore, to further increase the amount of PS deposited within the skin layers, AE PCs were combined with dissolving MNs. This approach led to a dual (i.e., MNs-PACT) smart (i.e., effective only in the infected tissue upon irradiation only) strategy to target the dermal infection locally while reducing possible systemic side effects. Hence, the MNs could enhance the deposited amount of AE in the infected targeted tissue and demonstrate no activity in dark conditions, showing promise for activation exhibited locally within infected skin areas only upon irradiation.

To further prove this approach, a concentration of 4 μg/mL was chosen for the PCs-MNs to be tested for the PACT in this study based on the effective concentration for the PCs before combining them with the MNs (cf., Figure 20). The data showed that incorporating the PS M-PCs and N-PCs within the MNs led to well-improved PACT properties, as shown in Figure 21. The AE N-PCs showed pronounced bacterial inactivation even with a low concentration (4 μg/mL) compared to that of the AE M-PCs (Figure 21).

**Figure 21:**
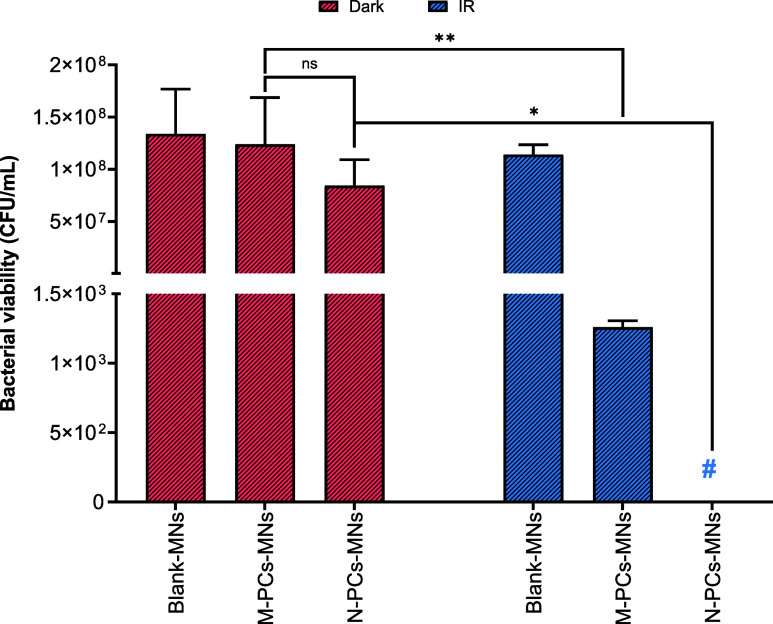
Bacterial viability of *S. saprophyticus* subsp. *bovis* on the obtained MNs containing M-PCs and N-PCs before (dark) and after (IR) irradiation. # indicates no detectable bacterial viability following irradiation. Blank MNs (i.e., without drug) were used as a control. MNs represent microneedles, M-PCs represent micronized PCs, and N-PCs represent nanosized PCs from AE (Aloe emodin). Data presented as means + SD, *n* = 4.

The improved PACT efficacy of the N-PC-MN system can be attributed to the enhanced solubility and dermal deposition achieved through nanosization. The smaller particle size of N-PCs increases the surface area and dissolution rate, resulting in a more extensive surfactant–polymer layer surrounding the solubilized AE molecules (Figure 22). This layer enhances PS availability and facilitates greater light absorption and ROS generation upon irradiation. The polymers used in MN fabrication further contribute to this effect by promoting additional solubilization of AE, thereby strengthening the photodynamic response. The nanoscale dimensions of N-PCs also enable deeper and more uniform penetration through MN-created microchannels, positioning the PS in closer proximity to bacterial cells within the skin. In contrast, the PACT of M-PCs did not significantly improve upon MN incorporation, likely due to their limited dissolution, reduced solubilization, and restricted diffusion within the skin layers (Figure 22). These findings are consistent with previous reports demonstrating improved activity of micronized or nanosized drugs incorporated into dissolving MNs.^67^ Furthermore, partial amorphization observed in the DSC and XRD analyses may influence the AE photoreactivity under irradiation. Amorphous domains generally enhance molecular mobility and light absorption efficiency, potentially increasing photoreactivity compared with the crystalline state. However, in the developed system, the amorphization was only partial and localized, and no significant change in AE photoreactivity was detected under the experimental irradiation conditions.

**Figure 22:**
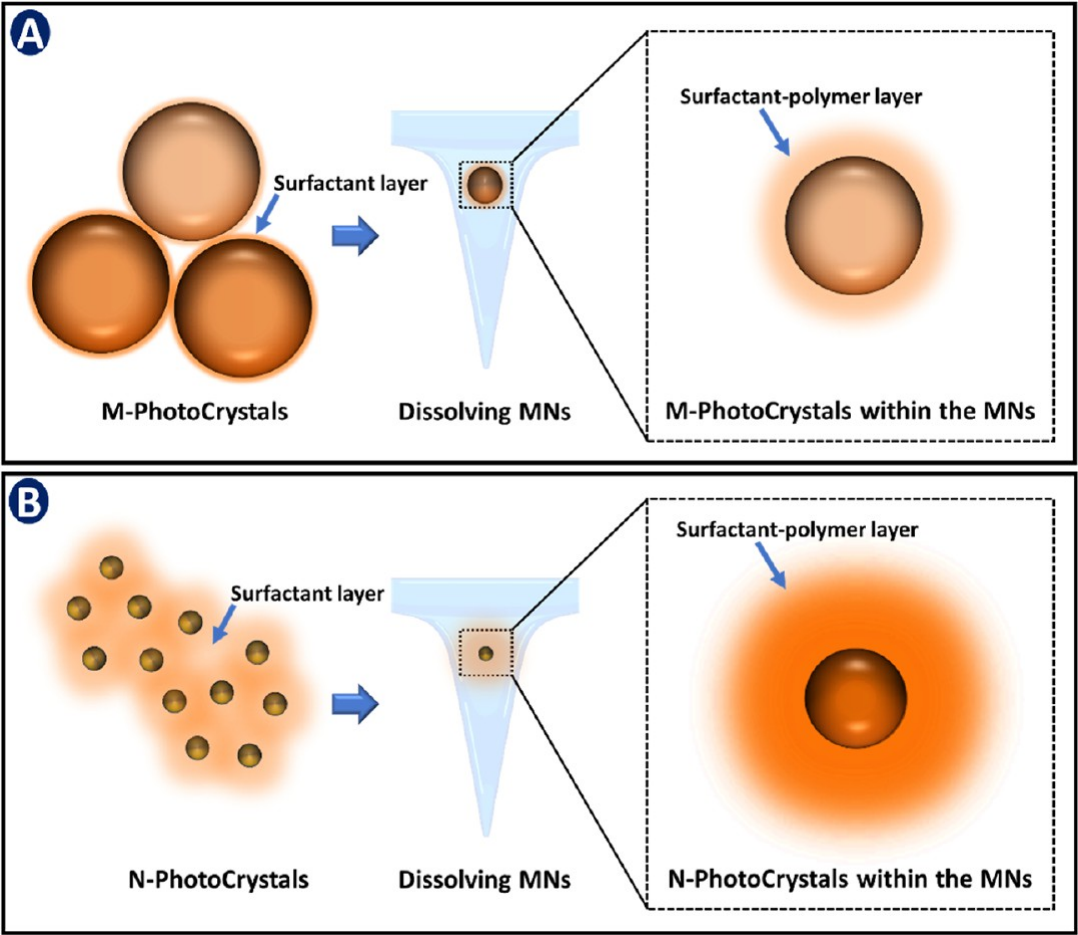
Illustration explaining the mechanism of the enhanced activity of PhotoCrystals following incorporation into microneedles (MNs), showing a higher amount of the surrounding surfactant–polymer layer of the N-PhotoCrystals (nano-PhotoCrystals; i.e., photosensitive nanocrystals) compared to M-PhotoCrystals (micro-PhotoCrystals) after MN integration. (A) M-PhotoCrystals incorporated within MNs. (B) N-PhotoCrystals incorporated within MNs.

### 3.4 Assessment of Biocompatibility and Irritation Potential

Since both the micronized and nanosized AE formulations show good skin deposition profiles that were further improved after combination into MNs, the aim of this study was to increase the amount deposited within the skin layers to treat ABSSTIs was achieved. To this end, it was necessary to study the irritation potential and biocompatibility of the formulations obtained. Therefore, HET-CAM was utilized to assess the irritation potential and evaluate the biocompatibility. This method was developed as an alternative to the Draize test to promote the principles of the 3Rs (replacement, reduction, refinement).

All four formulations, i.e., micronized and nanosized AE and the corresponding MNs, were analyzed regarding their irritation potential and biocompatibility using the HET-CAM model alongside blank MNs as a bench control. The blank MNs were biocompatible since they did not show any irritation (i.e., hemorrhage, lysis, or coagulation) using the irritation scoring system (Figure 23). Micronized formulations, both alone and when incorporated into MNs, induced irritation as evidenced by visible hyperaemia, characterized by a marked increase in the redness of blood vessels, and a slight increase in the overall vascular density in the chorioallantoic membrane. By contrast, the nanosized formulations before and after incorporation within the MNs did not show any irritation (Figure 23). This indicates the irritation caused in the micronized formulations was due to the bigger particle size in comparison to the nanosized formulation, as no irritation for the nanosized or the blank MNs was noticed. These data are in line with previously reported data on similar nanosized formulations.^68,69^ Thus, it can be concluded that nanosized AE PCs could increase dermal deposition in a safe manner, making the PS ready for PACT within the skin layers and avoiding any irritation.

**Figure 23:**
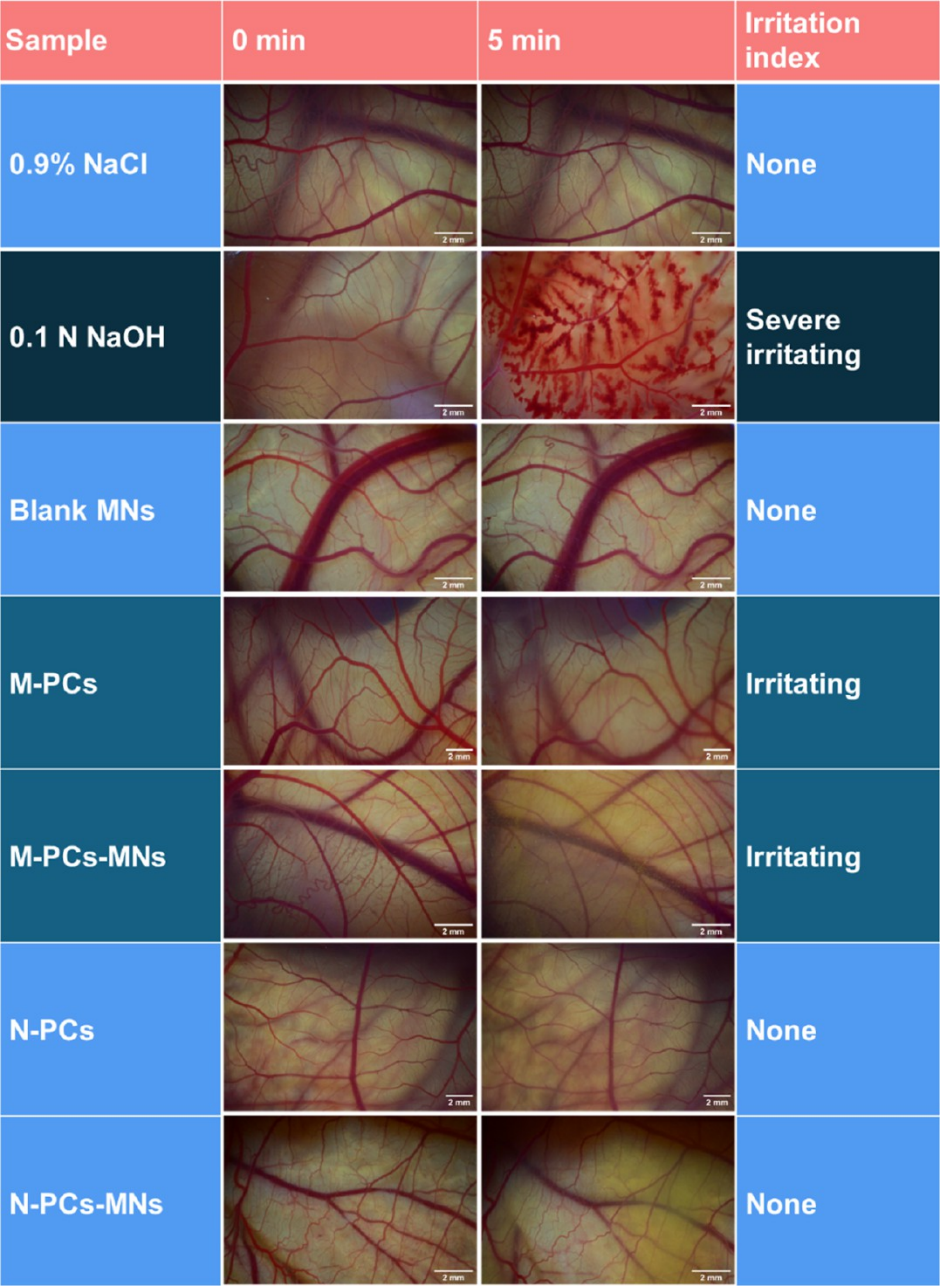
Hen’s egg test chorioallantoic membrane (HET-CAM) showing the biocompatibility and irritation potential of the tested formulations. 0.9% NaCl, 0.1 N NaOH, and blank MNs (without drug) were used as controls. The CAM micrographs were recorded at 0 and 5 min after applying the formulations and the irritation potential was observed. MNs represent microneedles, M-PCs represent micronized PCs, and N-PCs represent nanosized PCs from AE (Aloe emodin). Scale bars represent 2 mm.

Thus, photosensitive nanosized AE (i.e., nanosized PCs) and their dissolving MNs were successfully obtained and fully characterized regarding their physicochemical and mechanical properties, morphology, stability, skin permeation and deposition profile, PACT activity, and biocompatibility. The data obtained confirmed that AE could be nanosized using a wet milling method, and the obtained formulation showed good physicochemical characteristics that did not change upon fabrication of MNs. The dermal deposition was improved when the nano- and micro-AE PCs were incorporated into the MNs. Furthermore, the stability of the final formulations showed that AE was stable during the study time conducted. The PACT and biocompatibility of all the formulations proved that the PACT activity of the nanosized PCs was most promising when compared to the micronized PCs. Thus, the PCs designed in this study have shown potential in overcoming previously reported issues to improve the solubility, loading efficiency, and dermal delivery of the natural PS for skin infections. Furthermore, incorporation of this developed system within MNs was also successful as a dual targeting safe strategy to reach specific skin tissue by localizing the drug in the site of action using MNs, with activation of the drug after irradiation in the infected area. Ultimately, producing the PACT effect only on the infected area without disturbing the skin microbiome will prevent unnecessary exposure and the potential of developing AMR. Therefore, this study showcases a novel formulation principle for a safe and dual “smart” approach for the painless intradermal delivery of a natural hydrophobic PS to tackle AMR and efficiently treat ABSSTIs. Moreover, this system is capable of addressing the challenges linked to topically applied formulations. Unlike antibiotics that are administered orally or through intravenous injection, MNs can enhance the dermal delivery of AE for infections in soft and deep skin tissues, all while minimizing pain and avoiding complications associated with oral administration, such as drug localization in the infected skin tissue and possible disruption to the gastrointestinal microbiome.

## 4 Conclusions

Novel AE photosensitive M-PCs and N-PCs were reported and used for PACT for the first time. The integration of PCs with MNs provides the first matrix-free PS delivery system, eliminating carrier-related challenges associated with PS encapsulation within nanocarriers and potential integrity issues arising from MN–polymer matrix interactions (e.g., PS-loaded liposomes incorporated into MNs). This study illustrated a simple method to introduce the PS and an efficient drug delivery matrix-free system in which hydrophobic antibiotic PS enabled dermal deposition within the skin, with activity shown only after irradiation, which led to a specific and targeted effect in the desired infected tissue. Incorporating the micronized and nanosized PCs into MNs permitted a greater deposition of AE within the skin tissue and showed a better PACT for the nanosized PCs. The nanosized formulation and the corresponding MNs were shown to be biocompatible and did not result in any irritation for the soft tissues when compared to the micronized PCs. Ultimately, the new PhotoCrystal technology presented in this work is a simple technique that is easily scalable and leads to effective and safe PACT, to act as a targeted system. In addition, including the PCs within the MNs led to a dual-targeted system, which can deposit a drug only in the infected tissue after activation by irradiation. This could be beneficial as it may reduce the systematic effects, is unlikely to disturb the microbiome, and thus can tackle AMR issues. Further studies are now needed to investigate the efficiency of the developed “smart” dual targeting system with other PSs and the use of different polymeric MNs formulations. In addition, a more detailed safety assessment of the new smart system should be performed and combined with an in vivo study.

## Data Availability

Data supporting the results presented are available upon reasonable request.
